# Spatiotemporal climatic signals in cereal yield variability and trends in Ethiopia

**DOI:** 10.1038/s41598-025-23452-7

**Published:** 2025-10-23

**Authors:** Kidist Abera, Sebastian Gayler, Hans‑Peter Piepho, Thilo Streck

**Affiliations:** 1 Institute of Soil Science and Land Evaluation, Biogeophysics, University of Hohenheim, Stuttgart, Germany; 2 Ethiopian Institute of Agricultural Research, P.O. Box 2003, Addis Ababa, Ethiopia; 3 Biostatistics Unit, Institute of Crop Science, University of Hohenheim, Stuttgart, Germany

**Keywords:** Climatic variability, Climatic impact, Crop yields, Linear mixed-effects model, Crop management, Ethiopia, Environmental sciences, Climate sciences, Climate change, Plant development

## Abstract

Climatic variability and recurrent drought can strongly affect the variability of crop yield and are therefore frequently considered a risk to food security in Ethiopia. A better understanding of how crop yields vary in space and time, and their relationship to climatic and other driving factors, can assist in enhancing agricultural production and adapting to and mitigating the impacts of climate change. We applied a multiple linear regression model to examine the spatiotemporal climatic signal (air temperature, precipitation, and solar radiation) in the yields of the most important crops (maize, sorghum, tef, and wheat) over the period 1995–2018. An analysis of the climatic data indicated that growing season temperature increased significantly in most regions, but the trends in precipitation were not significant. The yields of maize, sorghum, tef, and wheat tended to increase across most crop-growing areas, particularly in the west, but was highly variable. The results highlight large spatial differences in the contribution of climatic trends to crop-yield variability across Ethiopian regions. The trends in climatic variability did not significantly affect crop yields in some areas, whereas in the main crop-growing areas, up to − 39.2% of yield variability could be attributed to the climatic trends. Specifically, the climatic trends negatively affected maize yields but positively affected sorghum, tef, and wheat yields. Nationally, the average impacts of climatic trends on crop yields was relatively small, ranging from a 3.2% decrease for maize to a 0.7% increase for wheat. In contrast, technological advancements contributed substantially more to yield gains, with annual increases ranging from 4.3% for wheat to 5.1% for sorghum. These findings highlight the dominant role of non-climatic drivers, particularly improved agricultural technology, in shaping crop yield trends. Our findings underscore the spatial heterogeneity of climate impacts on agriculture and highlight the critical importance of technological progress in enhancing crop productivity. They also provide actionable insights for designing crop- and location-specific adaptation strategies, and stress the need for integrated, climate-resilient development pathways in the region.

**Supplementary Information:**

The online version contains supplementary material available at 10.1038/s41598-025-23452-7.

## Introduction

Food security has become a global policy and is an important goal of sustainable development^1^. Global food demand is expected to double by the 2050s compared to 2009^2^. Climatic variability and weather extremes strongly affect systems of agricultural production^3,4^. These risks have threatened food security at both local and global scales^2^. Climate change is projected to increase climatic variability and extremes further and to have many effects on crop production^5–7^. Understanding how climatic factors and crop production are correlated at different spatial and temporal scales to identify challenges to food security and to implement adequate adaptive measures is thus important.

Crop production in sub-Saharan Africa is highly sensitive to variations in climate^5,8–11^, particularly in Ethiopia due to the vulnerability of the rainfed cropping system to climatic variability and climate change^12–15^. About 95% of crop production in Ethiopia is from rainfed agricultural systems, mainly by small-scale subsistence farming and mostly during the rainy season (June to September). The spatiotemporal variability in precipitation and associated droughts is cause of food insecurity in the country^14,16–18^. Various studies have found that crop failure due to frequent dry spells during the growing season is a common risk in Ethiopia^17,19–21^. For example, the lack of rain or the occurrence of dry spells during critical stages of crop growth (flowering and grain filling) led to a 40% reduction in yield in northern Ethiopia^19^. A study by von Braun^21^ stated that a 10% decrease in precipitation from the long-term average in the growing season decreased national food production by 4.4%.

The country has become warmer and drier in recent decades, and climatic variability has increased^22–25^. Air temperature has increased^26–28^, and precipitation has varied greatly interannually and intraseasonally^20,29–31^. Previous studies have investigated the impact of climatic variability on crop yield at global^4,32,33^ to regional^9,34–36^ scales. Some studies have examined the relationship between crop production and local climate in Ethiopia^23,27,37,38^, with some studies focusing on the links between crop-yield variability and climatic variation^20,39,40^. These studies relied on observations from a limited number of meteorological stations and data sets for yield, which restricts the generality of their findings. To the best of our knowledge, the covariation of climate and crop yield trends across Ethiopia based on data sets has not been examined in detail. Understanding the link between climate trends and the variability of crop yield is essential to accurately assess recent progress in increasing yield and the impact of future climate change on crop production.

A study by Alemayehu and Bewket^39^ in three districts of northern Ethiopia during 2004–2013 indicated that crop production was negatively affected by climatic variability. Bewket et al.^40^ reported that crop production during 1994–2003 was significantly correlated with rainfall in the Amhara region of Ethiopia. A simulation study by Yang et al.^12^ found that the combined effects of climatic factors contributed to a decreasing trend in wheat yield, an increasing trend in maize yield, and no clear trend in the yields of millet and barley across Ethiopia for 1979–2014. None of these studies quantified the contribution of changes in individual climatic factors to the variability and trends of crop yield. The influence of climate on crop yields has also not been adequately and explicitly documented at either the national or local level due to Ethiopia’s varied geography, topography, and climate. Identifying the relationships between climatic factors and the variability of crop yields across space and time, however, is important for developing and implementing local impact-based adaptive and mitigative measures.

Several studies have projected that future climate change will significantly increase climatic variability across Ethiopia. The frequency and intensity of extreme weather events are expected to escalate^14,28,41–43^. Although crop yields are already showing sensitivity to shifts in temperature and precipitation patterns, these climatic influences are occurring alongside non-climatic drivers, such as technological innovations, that tend to enhance agricultural productivity. Consequently, disentangling the complex interplay of climate and non-climate factors presents a major challenge in accurately projecting future crop yields in the country.

We employed a mixed-effects regression model to understand the spatiotemporal variability and trends in the yields of four main cereal crops (maize, sorghum, tef, and wheat) in response to climate (air temperature, precipitation, and solar radiation) and non-climatic (technological advancements) factors across administrative zones (AZs) and districts in Ethiopia for 1995‒2018. The specific objectives were to: (1) examine the spatiotemporal variability and trends of climatic variables and crop yield, (2) explore how observed variations in crop yields were related to climate variations, (3) compute the impacts of individual and combined climatic trends on the crop yield variability, and (4) investigate the contribution of non-climatic factors to the changes in crop yield variability across the major crop growing areas of Ethiopia. Unlike previous studies in Ethiopia that primarily investigated the effects of climatic variability on crop yields using limited spatial or temporal data, this study provides a comprehensive, high-resolution assessment of both climatic and non-climatic factors driving crop yield variability and trends. Employing a mixed-effects regression framework across administrative zones and districts from 1995 to 2018, we are able to separate and quantify the respective contributions of these factors. This integrative approach offers new insights into the spatial heterogeneity of yield responses, revealing that non-climatic drivers play a dominant role in many crop-growing areas, a finding that has been largely overlooked in the existing literature. The novelty of this study lies in its ability to disentangle and quantify the relative contributions of climatic and non-climatic factors using long-term, spatially detailed data, thereby offering actionable insights for climate-resilient agricultural planning in Ethiopia and similar agroecological contexts.

## Material and methods

### Study area

**Fig. 1:**
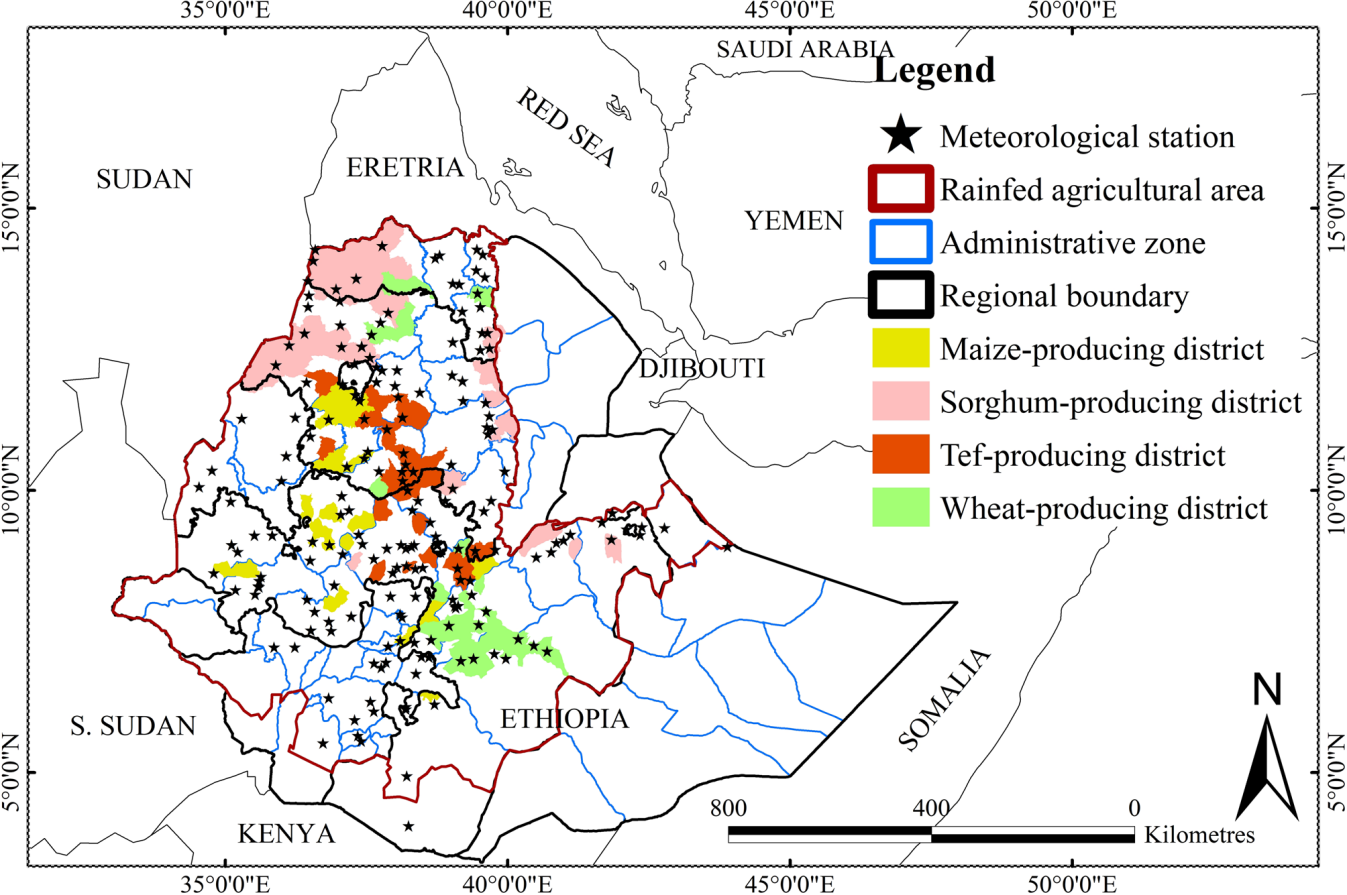
Map of the administrative zones and selected districts in Ethiopia used in this study. The 175 meteorological stations are represented by stars. A list of administrative zones, selected districts, and meteorological stations is provided in Supplementary Fig. 1 and Supplementary Table 1.

Ethiopia is in the eastern part of Africa and extends from 3 to 14° N and 33 to 48° E, with a total area of 1.13 million km^2^ (see Fig. 1 and Supplementary Fig. 1). The country has complex topography, with elevation ranging from 110 m below sea level at the Denakil depression in the northeastern lowlands to 4620 m a.s.l. at the peak of Mt. Ras Dejen in the northern highlands.

The climate of Ethiopia is strongly influenced by large-scale atmospheric circulations and orography^44^. The country has three seasons with different patterns of temperature and precipitation: the rainy, short rainy, and dry seasons. The rainy monsoon season (June to September) is the main source of rain for most parts of Ethiopia^44^. The dry season (October to January) is the coldest season, and the short rainy season (February to May) has light rain and the warmest months over most of the country.

Mean annual precipitation in 1995–2018 varied from 270 mm in the northeast to > 1257 mm in the west and southwest. Precipitation in the growing season (June to September) averages 730 mm. The mean air temperature ranges from 3.9 °C in the highlands to > 31.2 °C at the eastern periphery (see Supplementary Fig. 2 and Table 1).

The most common farming systems in the country are mixed agricultural systems and pastoralist systems. The majority of the population resides in the humid and sub-humid highlands and practices mixed crop-livestock agriculture. Pastoralism is practiced in the arid and semi-arid lowlands. The dominant cultivated crops are cereals, accounting for about 81.5% of the total cultivated crop area and 88.5% of total grain production^45^. Tef (*Eragrostis tef* (*Zuccagni*) *Trotter*) (24.1%), maize (*Zea mays* L.) (17.7%), sorghum (*Sorghum bicolor* (L.) *Moench*) (14.2%), and wheat (*Triticum spp*.) (13.9%) are the most widely planted cereals.

Ethiopia is divided into 11 regional states and two chartered cities (Fig. 1)^45^. Regional states are subdivided into Administrative zones (AZs), forming a second level of subdivision. The number of AZs per region varies, but most regions have 5–12 AZs; Oromia, the largest, has > 21 AZs. The total number of AZs is 75. The AZs are subdivided into districts (*woredas*), with each AZ having about 15 districts. Districts are crucial administrative units for agricultural decisions, so district data for agricultural production are essential for planning, implementing, and evaluating agricultural interventions. The two structural elements, AZs and districts, will serve as the basis for evaluating the data in this study.

### Data sources and processing

We used crop yield and climate data for the period 1995–2018 (Table 1). Climatic variables included daily and monthly records of minimum, maximum, and mean air temperature and total precipitation from 175 meteorological stations provided by the Ethiopian National Meteorological Agency (NMA) (Fig. 1 and Supplementary Table 1 for station distribution). All station data underwent a standardized quality control protocol to identify and correct outliers, temporal inconsistencies, and missing observations. To address data gaps, we employed high-resolution reconstructed gridded datasets developed by the NMA. These gridded products integrate ground-based observations with spatial interpolation techniques to ensure temporal coherence and continuity across Ethiopia’s heterogeneous landscapes^39^.

**Table 1:** Information for the data used in this study.

Data	Source	Unit	Description	Data processing
Temperature	Ethiopian National Meteorological Agency	°C	Daily and monthly minimum, maximum, and average temperatures for 175 stations	Gridded at a resolution of 1 km using inverse distance weighing interpolation and aggregated at administrative zone and district levels
Precipitation	Ethiopian National Meteorological Agency	mm	Daily and monthly total precipitation for 175 stations	Gridded at a resolution of 1 km using inverse distance weighing interpolation and aggregated at administrative zone and district levels
Solar radiation	National Centre for Environmental Prediction (NCEP, Zhao and Running, 2010)	W/m2	Monthly total solar radiation at a resolution of 0.5°	Disaggregated to a resolution of 1 km using bilinear interpolation, and aggregated at administrative zone and district levels
Crop yield	Ethiopian Central Statistical Agency	kg/ha	Main rainy-season yields for 1995–2018	Arranged for maize, sorghum, tef, and wheat crops at administrative zone and district levels

Crop yield data for maize, sorghum, tef, and wheat in the rainy season were obtained from the Ethiopian Central Statistical Agency (CSA) for 75 AZs and 541 districts. To address yield data quality, we excluded AZs in the northeastern and southeastern lowlands where cultivation is limited or absent during the rainy season. Furthermore, district-level analyses focused on 25 productive districts identified by Warner et al.^46^ with relatively complete records (≥ 21 years) and < 10% missing values (Supplementary Table 2). Where district-level yield data were missing or unreliable, we substituted these with corresponding AZ-level values from the CSA, following a hierarchical imputation strategy^47,48^. This substitution was limited to maintain spatial resolution while minimizing the propagation of uncertainty. Yield records used in this study ultimately spanned 57 AZs for maize, 62 for sorghum, 51 for tef, and 44 for wheat (see Supplementary Methods for more detail). Detailed documentation of CSA’s crop yield data collection methodology, which relies on a stratified random sampling approach with field-level measurements, is provided in the Supplementary Methods.

To align the station-based climate data with the spatial resolution of yield data at AZs and district levels, we applied Inverse Distance Weighting (IDW) interpolation to generate continuous gridded surfaces (1 km resolution) for monthly mean temperature and total precipitation. IDW is a deterministic geostatistical method that assigns greater influence to nearby observations, thereby preserving local climatic variability while reducing the influence of distant data points. The resulting interpolated grids were aggregated to the AZ and district levels for subsequent analyses focused on the main growing season (June to September). The performance of the interpolated datasets was assessed through validation against independent observed station data for the 2010–2018 period (see Supplementary Table 5 in the Supplementary Methods). Results demonstrated strong agreement, with high correlation coefficients and low error rates, thus confirming the suitability of the gridded datasets for spatiotemporal yield-climate assessments at the sub-national scale.

Monthly solar radiation data were obtained from the DOE-II reanalysis dataset of the National Centre for Environmental Prediction (NCEP) at a spatial resolution of 0.5°^49^. These data were downscaled to 1 km resolution using bilinear interpolation. We acknowledge that, as modeled reanalysis products, the solar radiation estimates may introduce spatial uncertainty, particularly in topographically complex regions, and this limitation should be considered when interpreting solar radiation effects on crop productivity.

Spatial averaging of the gridded climate variables data at the AZ and district levels was conducted using the zonal statistics tool in ESRI ArcMAP 10.2 (https://www.esri.com/en-us/arcgis/products/arcgis-desktop/resources). These procedures resulted in a harmonized, quality-controlled dataset of growing season mean temperature, total precipitation, and solar radiation for the period 1995–2018, fully consistent with the crop yield data used in the analysis.

### Determining trends and variabilities

**Fig. 2:**
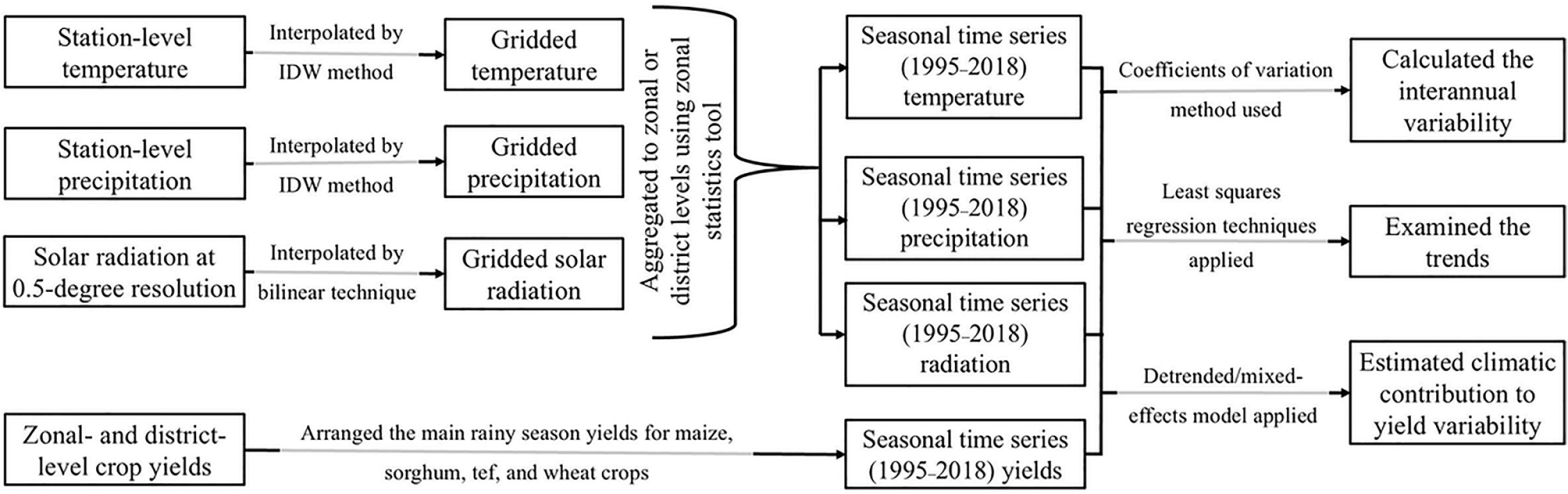
Methodological procedure for evaluating how the interannual variability in crop yield is correlated with the trends in climatic and non-climatic factors. The data sets were gridded at a resolution of 1 km × 1 km and then aggregated to administrative zonal and district levels in seasonal time series of growing season means for 1995–2018. IDW: Inverse Distance Weighting.

Procedures for evaluating the data are presented in Fig. 2. Growing season (June to September) data on mean air temperature, total precipitation, and total solar radiation, aggregated at the AZ or district level, were used. The interannual variability of climatic factors and crop yields across the study period (1995–2018) was quantified using coefficients of variation (CVs). The CV, a dimensionless metric, provides a standardised measure of variability for individual variables (see Supplementary Methods for more detail). The CV was computed without correcting for the trend. In addition, we report standard deviations (SDs) alongside mean yield values at the national level for descriptive purposes.

Trends of crop yield, mean air temperature, and total precipitation during the growing season were calculated using linear regression (see Supplementary Methods). A simple linear regression model is a widely used statistical approach for detecting linear trends over time^4,50,51^. This model is fitted using the least squares method, ensuring an optimal linear representation of the data. The resulting trend is expressed as a straight line that best approximates the observed values. Trends were considered significant at the 5% level. In addition to the AZ-levels analysis, linear trends for growing season temperature and precipitation were also calculated at the individual meteorological station level (n = 175) using linear regression, and the results are summarized in Supplementary Table 1.

We performed a further analysis using AZ-specific first-difference regression models to relate the year-to-year variability of yield to the year-to-year variability of climate variables (see Supplementary Methods). A multiple linear regression was performed for each AZ, with the intercept of the regression representing the average change in yield per year with climate held constant^51^. The obtained regression coefficients imply the percent of change in yield per unit of weather change (e.g., the % change in yield per 1 °C increase). The coefficient of determination (*CD*) from these first-difference regression models was used to assess the extent to which climatic variability explained the variability in crop yield across the AZs.

### Estimating climatic impacts on crop yield variability and trends

To assess the contributions of climatic trends to the observed crop yield trend (Fig. 2), we applied a linear mixed-effects model with random intercepts and random slopes as well as an autoregressive term to each of the four crops (Eq. 1 and see Supplementary Methods for more detail). Our approach accounts for both fixed effects (mean regression on climatic variables) and random effects (differences between AZs). The year, temperature (T), precipitation (P), radiation (R), and their quadratic terms (T^2^ and P^2^) were modelled as the fixed-effect variables, whereas the AZs were treated as random-effects variables. The model recognises that observations within the same AZ are more likely to be similar than those from different AZs. An unstructured covariance matrix was imposed to model the correlation between random intercepts and random slopes for the temporal trend and climatic variables, thus making the models translationally invariant^52^. To account for temporal autocorrelation, we included a first-order autoregressive (AR1) serial correlation structure in the models. We also tested interaction terms between T and P (TP), between T and R (TR), between P and R (PR), as well as the three-way interaction (TPR). However, these interactions did not significantly enhance model performance or explanatory power and were therefore excluded from the final model. The model is given by:

1 $${Log(Y}_{i,t})={\beta }_{0} + {b}_{0i}+(\zeta + {z}_{i})*{year}_{t}+ \sum_{j=1}^{J}\left({\beta }_{j}+ {b}_{j,i}\right){X}_{j,i,t}+{\varepsilon }_{i,t}$$

where ${Log(Y}_{i,t})$ represents the logarithm of yield for AZ *i* in year *t*, ${\beta }_{0}$ is the global intercept, ${b}_{0i}$ is the random intercept for each AZ, ${z}_{i}$ is the random AZ-specific slope for the temporal trend (year) that adds variation to the fixed effect, $\zeta ,$ of time in the AZs, the term ${\beta }_{j}+{b}_{j, i}$ contains coefficients for the ${j}{th}$ climatic predictor variable ${X}_{j,i,t}$ (i.e. T, ${\text{T}}^{2}$, P, ${\text{P}}^{2}$, and R), composed of a fixed effect representing the average response to the climate variable, ${\beta }_{j}$, and a random effect, ${b}_{j, i}$, accounting for the variability in the effect of climate in the AZs. ${\varepsilon }_{i,t}$ (Eq. 2) is the error term, which was assumed to have an AR1 structure that allows identifying and accounting for temporal autocorrelation within the AZs.

2 $${\varepsilon }_{i, t} = \rho {\varepsilon }_{i,t-1}+ {\eta }_{i,t}$$

where ρ is autoregressive parameter, and η is independent noise term.

Crop production data typically follows a log-normal distribution^4^. To better align with this distribution, we apply a log transformation to yield ${log(Y}_{i,t})$. This transformation helps normalize the data, ensuring it meets a key assumption of linear regression models. Additionally, the log specification enhances variance stabilization, interpretability, and overall model fit (see Supplementary Fig. 10 in the Supplementary Methods). Specifically, the transformation ensures positive predicted responses on the original scale. Random intercepts across AZs account for variance caused by omitted AZ-specific factors in the model, such as soil quality and management practices^4^.

The addition of quadratic terms for temperature and precipitation in the model serves to encompass the nonlinear impact of meteorological conditions on crop yields^4,7,32^. The statistical significance was assessed at the 5% level. When a quadratic term was found to be significant, we included the corresponding linear term, even if they were not statistically significant, to preserve the model’s structural integrity. According to the principle of marginality, higher-order terms (e.g., T^2^ or P^2^) should not be included in a model without their respective lower-order terms (e.g., T or P). Omitting these linear terms could lead to biased coefficient estimates for the quadratic terms, potentially distorting their interpretation. While T and P may not always exhibit strong statistical significance in this mixed-effects model, their inclusion ensures mathematical coherence, biological relevance, and interpretability while maintaining the hierarchical structure necessary for accurate inference^53^. To ensure model stability and reliable coefficient estimates, we opted to use mean T as a consolidated and statistically robust temperature indicator. We conducted a sensitivity analysis in which minimum and maximum temperatures were included as separate covariates in the model (see Supplementary Fig. 11 in the Supplementary Methods). The results from this alternative specification were consistent with those obtained using mean temperature.

All predictor variables were standardized to eliminate dimensional inconsistencies. Finally, to account for differences in the impacts of non-climatic factors across AZs, such as technological improvements, we included both time (*year*) and AZs in the mixed-effects regression model (Eq. 1).

Climatic variables such as *T*, *P*, and *R* can also have long-term trends due to ongoing climate change. To analyse the effect of climatic trends we followed the approach by^4^, detrended time series of climatic variables were calculated from the observed time series by subtracting predicted values obtained from random intercepts and random slopes regression model and adding the initial value (1995 in this study), as in Eqs. (3) and (4).

3 $${X}_{d,i, t}=\left({X}_{obs, i, t}-{X}_{p, i,t}\right)+{X}_{1995}$$

4 $${X}_{p,i,t}={X}_{i}+{m}_{i}\cdot ({year}_{t}-1995 )$$

where ${X}_{d,i, t}$ is the detrended value of *X* (*T*, *P*, and *R*) in administrative zone *i* and year $t$, (${year}_{t}$ =1995, …, 2018), ${X}_{obs, i, t}$ is the observed value in AZ *i* and year $t$, and ${X}_{1995}$ is the initial value of the time-series. The predicted value for year $t$, ${X}_{p,i,t}$, was calculated using the model given in Eq. 3, where ${X}_{i}$ is the random intercepts and ${m}_{i}$ the random slopes (trend). Note that our models are invariant to translations in Eqs. (3) and (4) because we fit an unstructured variance–covariance matrix for random intercepts and slopes.

We evaluated five scenarios to estimate the impacts of the climatic trends on crop yield. These scenarios involved different combinations of observed and detrended variables for temperature (*T*), precipitation (*P*), and solar radiation (*R*). The model was used to predict yields for each crop in each AZ for 1995–2018 as: (i) observed *T*, observed *P*, and observed *R*, (ii) observed *T*, detrended *P*, and detrended *R*, (iii) detrended *T*, observed *P*, and detrended *R*, (iv) detrended *T*, detrended *P*, and observed *R*, and (v) detrended *T*, detrended *P*, and detrended *R*. We evaluated the individual contributions of the trends in *T*, *P*, and *R* to the overall trend in yield (all trends in kg per year) from the differences between scenarios (i) and (ii), (i) and (iii), and (i) and (iv), (i) and (v), respectively, and expressed the trends relative to the trend of scenario (i), separately for each AZ:

5 $$Climatic\,\, trends\, impact= \left(\frac{trend \,of \,scenario\left(i\right)-trend \,of\, scenario(v)}{trend \,of\, scenario(i)}\right) x 100$$

To illustrate the impacts of climate trends on crop yield trends at a finer scale than the AZ scale, we conducted a further linear mixed-effects model analysis at selected potential district levels using observed district yield data.

### Relating crop yield variability and trends to non-climatic factor

Crop yields are influenced by climate change and variability, but also by non-climatic factors such as practices of crop management and technological advances. These non-climatic factors often vary across AZs due to differences in resource availability, developmental priorities, and farming systems. Our mixed-effects regression model (Eq. 1) accounts for these variations by including a general temporal trend for each AZ. The temporal trend (*year*) serves as a composite proxy for technological advancements, including the adoption of improved cultivars, enhanced input use (fertilizers, pesticides), irrigation expansion, and better agronomic practices. These factors collectively drive yield trends independent of climatic variability^19,42,54^. By including this trend as a fixed effect, the model separates the effects of climate-related changes from those driven by technological developments. While explicit data on these factors are unavailable at the AZ level, the further inclusion of random effects allows the model to capture the local variation around the overall trend line.

It is important to note that this non-climatic trend may also capture a minor influence from rising atmospheric CO_2_ concentrations. However, previous research suggests that this effect is relatively small compared to the yield gains attributed to technological improvements^4,55^.

## Results

### Variability and trends of climatic factors

**Fig. 3:**
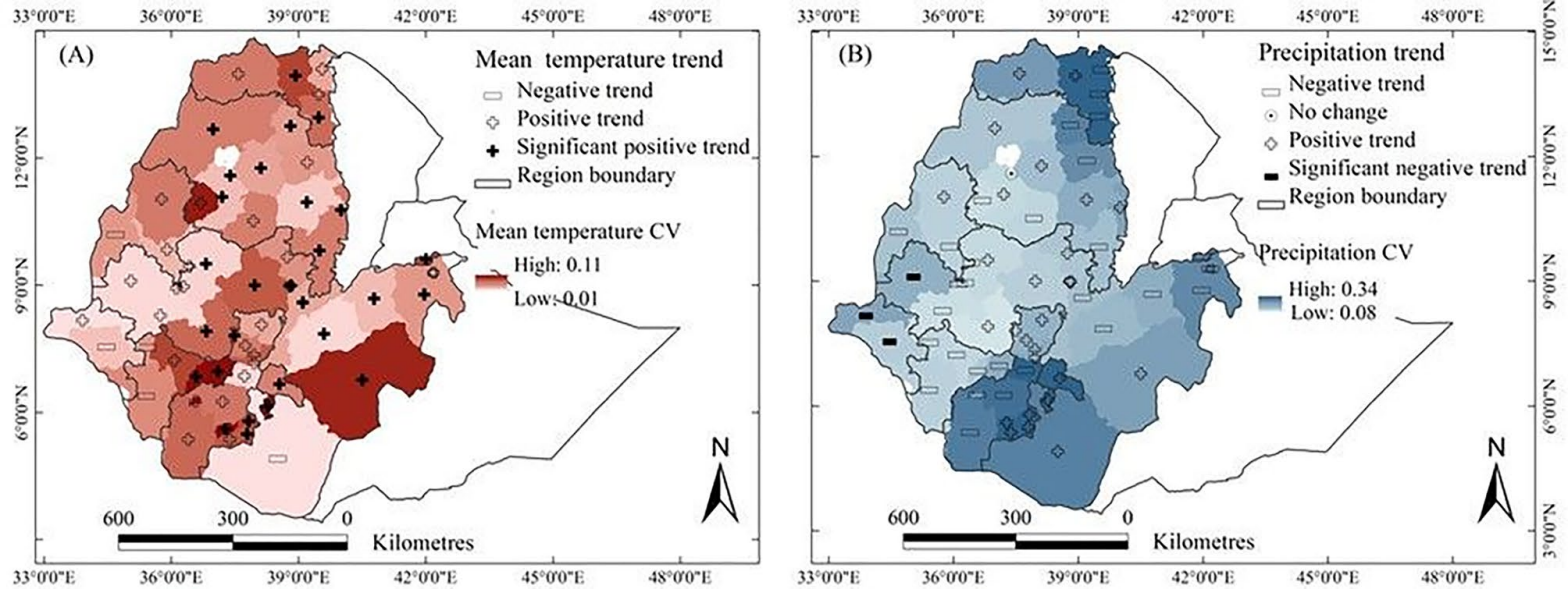
Coefficient of variations (CVs) and trends for growing season average air temperature (**A**) and total precipitation (**B**) for the administrative zones in the main crop-growing areas of Ethiopia 1995–2018. White zones denote areas where the crops of interest are not grown or where no data were available. Trends are represented by ‘ + ’ for increases, ‘ − ’ for decreases, and ‘⊙’ for no change, where the bold type indicates that the trend was significant at the 95% confidence level. The value of the CV is indicated by the colour scale.

The spatiotemporal variabilities and trends of temperature and precipitation during the main growing season (1995–2018) are presented in Fig. 3 (see also Supplementary Table 3). Temperature variability, expressed as the coefficient of variation (CV), showed distinct spatial patterns across AZs (Fig. 3A). The highest variability (CV up to 0.11) occurred in parts of northeastern and southeastern Ethiopia, particularly in Central Tigray, Bale, and Borena AZs. In contrast, temperature was the least variable (CV as low as 0.01) in the western areas. Variability of temperature was generally higher in the most recent years (late 2000s and 2010s) than in former periods (see Supplementary Figs. 3 and 4).

Temperature trends were largely positive across the country during the growing season (see Supplementary Tables 1 and 3 for weather stations and AZs, respectively). Warming rates ranged from + 0.83 °C per decade in AZs such as Central Tigray, West Gojjam, and Dawuro, to a cooling of − 0.58 °C per decade in Borena and West Omo. The national average warming rate was + 0.31 °C per decade. These increasing trends were statistically significant (*p* < 0.05) in 57% of AZs and 65% of weather stations.

Precipitation variability also showed considerable spatial heterogeneity (Fig. 3B). The highest CVs (up to 0.34) were recorded in the southwestern and eastern parts of the country, including AZs like Eastern Tigray, Gamo, and parts of Eastern Oromia. In contrast, the northwestern and central highland AZs showed relatively low variability (CV as low as 0.08). Since the 1990s, precipitation variability has increased, especially in eastern and southern Ethiopia, marked by more frequent and intense rainfall anomalies (see Supplementary Figs. 3 and 4).

Precipitation trends, however, were more spatially variable and less consistent than temperature. The overall national trend in growing season precipitation was a decline of − 47.5 mm per decade. The highest positive trend (up to + 42 mm/decade) was observed in some western AZs, while the strongest negative trend (− 89 mm/decade) occurred in southeastern Ethiopia. Notably, no AZ exhibited a statistically significant positive precipitation trend. Three AZs showed significant negative trends. At the station level, 13 weather stations had significant declines, while eight stations showed negative but non-significant trends (see Supplementary Table 1).

In contrast to the broadly consistent and significantly increasing temperature trends, precipitation trends were more spatially fragmented and statistically weaker. This contrast suggests that while temperature is rising uniformly across Ethiopia, precipitation patterns are more complex. Importantly, areas experiencing negative precipitation trends often coincided with positive temperature trends, such as in Borena, Bale, and Eastern Oromia, indicating a compounding effect of warming and drying. Conversely, AZs with relatively stable or increasing precipitation, mainly in the west and southwest, tended to have lower temperature variability and more stable climates.

### Variability and trends of crop yields

**Fig. 4:**
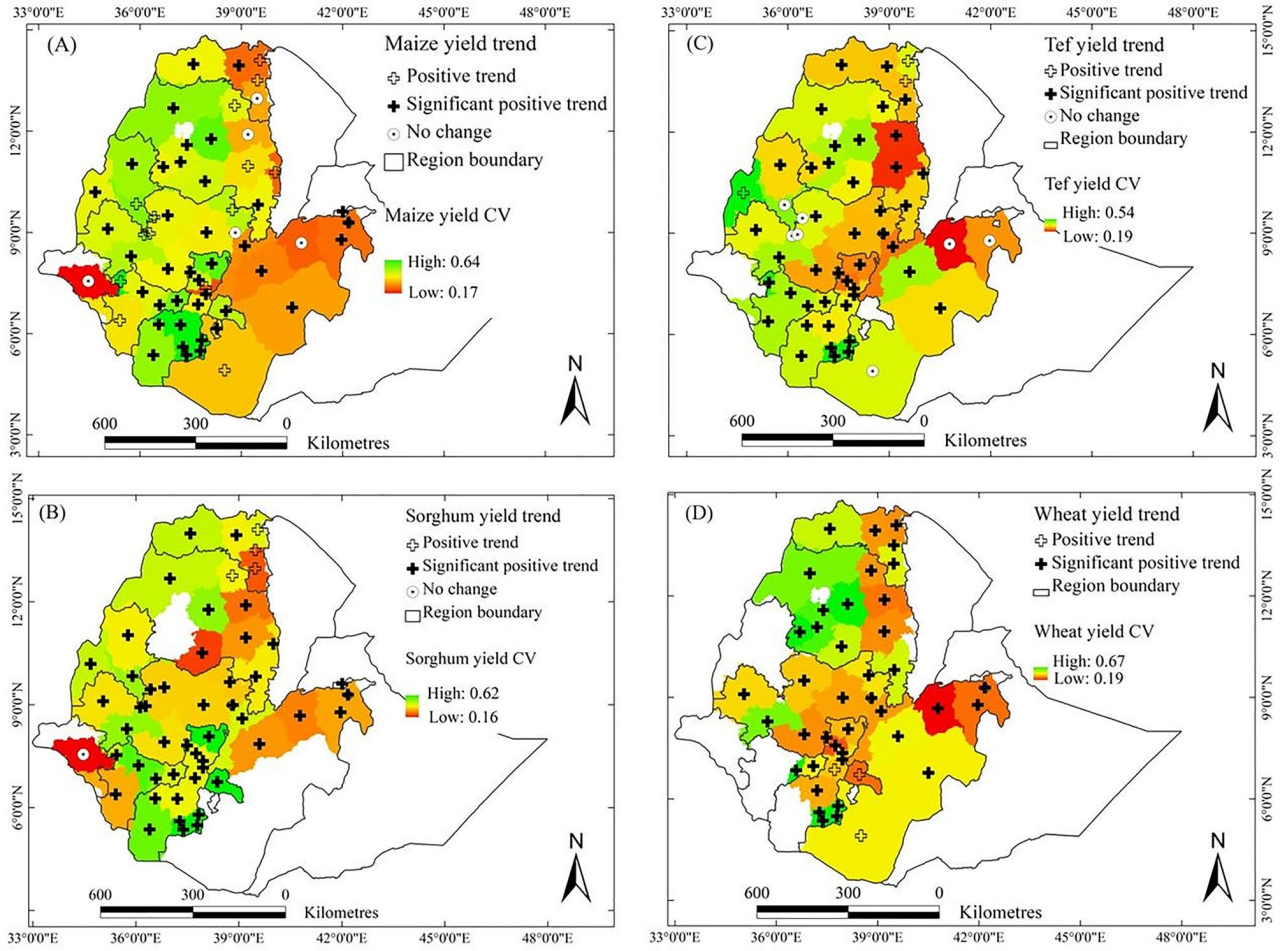
Trends and coefficients of variation (CVs) of the yields of maize (**A**), sorghum (**B**), tef (**C**), and wheat (**D**) for the administrative zones in the main crop-growing areas of Ethiopia for 1995–2018. White zones denote areas where the crops of interest are not grown or where no data were available. Trends are represented by ‘ + ’ for increases and ‘⊙’ for no change, where administrative zones with bold plus signs indicate that the trends were significant at the 95% confidence level. The value of the CV is indicated by the colour scale.

The interannual variability (CV) and trends in the yields of maize, sorghum, tef, and wheat were assessed across AZs in Ethiopia for the period 1995–2018 (Fig. 4, see also Supplementary Figs. 3 and 5). Additional analyses were conducted at the district level for 25 high-productivity areas identified by Warner et al.^46^, where consistent yield data were available for all four crops (Supplementary Table 2).

Maize yield variability (CV range: 0.17–0.64) was moderate to high across AZs (Fig. 4A). The lowest variability was observed in the high-yielding AZs of East Gojjam, North Gondar, West Wollega, and North Shewa. In contrast, higher variability occurred in parts of central and southern Ethiopia. A positive yield trend was detected in the majority of AZs, with many statistically significant increases particularly in western and southwestern regions. No AZ experienced yield declines. On a national scale, maize yields increased by an average of 154.4 kg/ha/year during the study period. At the district level, maize yield variability also varied substantially, with the highest variability recorded in Boset, Bore, Alaba, and Dembecha. Of the 25 high-productivity districts, 15 showed statistically significant upward trends in maize yields (*p* < 0.05), while only one district experienced a decline.

Sorghum showed relatively high interannual variability, with CVs ranging from 0.16 to 0.62 (Fig. 4B). The northwestern and southern regions displayed particularly higher variability than in other areas of the country. Despite this, the average level of variability was comparable to that of maize. Positive yield trends were observed across nearly all AZs, with significant increases in parts of eastern and northern Ethiopia, notably in West Gojjam, which recorded the highest rate at 137 kg/ha/year. The national average annual increase of Sorghum was 94.9 kg/ha/year. At the district level, variability was highest in Meiso, Chilga, Kalu, and Gondar Zuriya. Nevertheless, 13 of the 25 districts experienced statistically significant increases in sorghum yield over time.

Tef had the lowest interannual yield variability among the four crops, with CVs ranging from 0.19 to 0.54 (Fig. 4C). Its yield variability was especially low in the western regions, suggesting more stable yields over time. Tef yields increased in almost all AZs, with positive trends observed across the crop-growing areas. Several AZs in central and northern Ethiopia, such as East and West Gojjam and North Shewa, showed significant yield improvements. The average national increase in tef yield was 60.6 kg/ha/year. At the district scale, although tef generally showed lower variability compared to maize and sorghum, relatively high variability was noted in Becho, Dejen, Lomme, and Dera. Positive trends were nonetheless widespread, with 11 districts showing statistically significant improvements in yield.

Wheat yield variability was the highest among all crops (CV range: 0.19–0.67), with elevated variability in eastern and southern AZs (Fig. 4D). Despite this, wheat yields increased consistently across most AZs, with the strongest and most statistically significant trends occurring in Western Tigray, Arsi, Bale, and Awi. Western Tigray recorded the highest increase, at 184 kg/ha/year, while the national average wheat yield increased by 97.3 kg/ha/year. At the district level, wheat yield variability was most pronounced in Sinana, Gimbichu, Wegera, and Baso Liben. Nonetheless, wheat yield trends were predominantly positive, with 14 districts showing statistically significant increases over the study period.

At the national level, average crop yields over the study period were 2799 kg/ha for maize, 2020 kg/ha for sorghum, 1317 kg/ha for tef, and 2068 kg/ha for wheat (Supplementary Fig. 5). The corresponding variabilities (standard deviations, SDs) were 753.2 kg/ha, 455.3 kg/ha, 290.8 kg/ha, and 474.5 kg/ha, respectively. From 1995 to 2018, national yield levels increased by 27% for maize, 22% for sorghum, 26% for tef, and 24% for wheat, indicating a generally upward trend across crops and time (Supplementary Fig. 6).

Overall, yield variability and trends revealed consistent geographic patterns. Western Ethiopia tended to exhibit lower variability and stronger positive trends, particularly for maize and wheat. In contrast, eastern and southern regions experienced higher interannual variability, especially for sorghum and wheat, although yield trends in these areas remained positive in most cases. Across the 25 high-productivity districts, maize and wheat displayed widespread and statistically significant improvements, while sorghum and tef also showed positive trends, albeit with greater variability in spatial distribution and year-to-year performance.

### Covariation of crop yields and climatic factors

Climate factors often exhibit substantial interannual variability, making it essential to evaluate the sensitivity of crop yields to these climatic drivers. Sensitivity metrics provide valuable insights into how crop yields respond to unit changes in climate variables. Table 2 summarizes the average sensitivity of crop yields to growing season temperature, precipitation, and solar radiation based on first difference regression analyses.

**Table 2:** Average sensitivity of maize, sorghum, tef, and wheat yields to interannual variability in growing season temperature (*T*), precipitation (*P*), and solar radiation (*R*), based on first-difference regression models across administrative zones. Sensitivities are reported as the percentage change in yield per unit increase in each climate variable. The coefficient of determination (*CD*) represents the proportion of interannual yield variability explained by climate variability for each crop.

Crop	*T* (% per °C)	*P* (% per mm)	*R* (% per W/m^2^)	*CD*
Maize	− 0.25	− 0.0015	− 0.002	0.44
Sorghum	0.05	0.0016	− 0.007	0.39
Tef	0.19	0.0012	0.009	0.41
Wheat	0.34	− 0.001	0.002	0.45

The results indicate that maize yields are adversely affected by temperature increases. A 1 °C rise in growing season temperature corresponds to a 0.25% decrease in maize yield. This suggests that maize is particularly vulnerable to heat stress, likely due to its narrow optimal temperature range. In contrast, sorghum, tef, and wheat exhibit positive sensitivities to temperature, with yield increases of 0.05, 0.19, and 0.34% per °C, respectively. This indicates that these crops may benefit from moderate warming, potentially due to improved physiological activity or extended growing seasons in cooler highland areas.

Sensitivity to precipitation varies considerably across crops. Sorghum and tef show positive responses to increased precipitation, with sensitivities of 0.0016 and 0.0012% per mm, respectively. These findings suggest that these crops are more water-efficient and capable of utilizing additional rainfall to improve yields, especially in semi-arid regions. In contrast, maize and wheat show negative precipitation sensitivities (-0.0015 and -0.001% per mm), suggesting that excessive rainfall may either increase susceptibility to disease or waterlogging or reflect the timing mismatch between rainfall and critical crop stages.

The influence of solar radiation also differs markedly among crops. Increased radiation negatively affects maize and sorghum yields, with sensitivities of − 0.002 and − 0.007% per W/m^2^, respectively. This suggests that higher radiation levels may exacerbate evapotranspiration stress or lead to heat damage, especially in already warm environments. On the other hand, tef and wheat exhibit positive responses to increased radiation (0.009 and 0.002% per W/m^2^, respectively), suggesting that these crops are more capable of converting increased solar energy into biomass and yield under favourable moisture and temperature conditions.

**Fig. 5:**
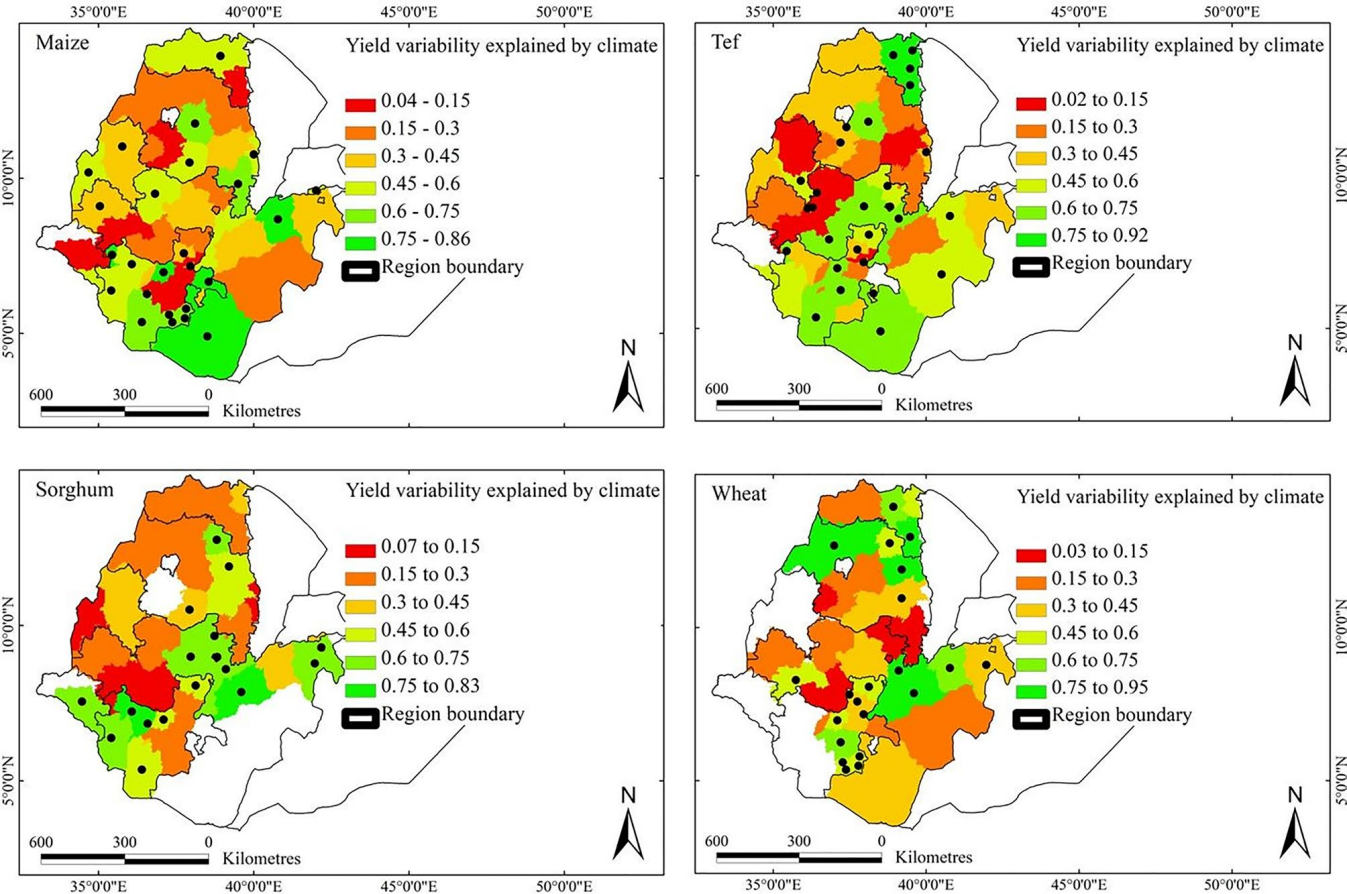
Variability of maize, sorghum, tef and wheat yields over 1995–2018 due to climatic variability in the main growing areas of Ethiopia. The values range from 0 to 1. A value of 1.0 implies that the entire variability was explained by climatic variability (*CD*, coefficient of determination). Dots denote the administrative zones where the relationship between climate and crop-yield variability is significant at the 95% confidence level. White zones denote areas where the crops of interest are not grown or where no data were available.

We evaluated the extent to which climate accounted for the variability of yield to further illustrate the relationship between observed variations in crop yields and climatic variations. This extent is represented by the coefficients of determination, *CD*, within first-difference models specific to each AZ, highlighting the proportion of crop-yield variability that climatic variability could explain (Fig. 5). Climatic variability accounted for significant variations in crop yields across the main growing areas, encompassing 45, 40, 49, and 37% of the areas for growing maize, sorghum, tef and wheat, respectively. These areas collectively represented 41, 33, 54, and 43% of the average production of maize, sorghum, tef and wheat in the country, respectively.

Climatic variability notably tended to account for a high proportion (> 60%) of the variations in maize yields, predominantly in the southern regions. The variability of sorghum yield due to climate was notably high (> 40%) across the growing areas, particularly in the south and southeast. The highest variability in tef yields associated with climate variability was primarily observed in the southern and northeastern growing regions. Similarly, interannual climatic variability accounted for a substantial portion of the variability in wheat yields, particularly in the northern and central parts of the main growing areas. On average, approximately 61, 63, 58, and 60% of the variations in maize, sorghum, tef, and wheat yields, respectively, were attributed to climatic variability amongst the AZs with significant relationships. Yet the extent to which climatic variability explained crop-yield variability varied greatly depending on specific locations and crops.

### Impact of climatic trends on crop yield trends

#### Role of climatic factors in yield trends

The impacts of individual climatic factors on crop yield trends over the period 1995–2018 were assessed using a linear mixed-effects regression model (Eq. 1). The yield impact (%) of each individual climatic factor was quantified as the ratio of the yield trend attributed to that factor to the overall yield trend in each AZ. This metric provides insight into the relative contribution of each climatic factor to the yield trend in a given AZ compared to the overall AZ-specific yield trend.

**Fig. 6:**
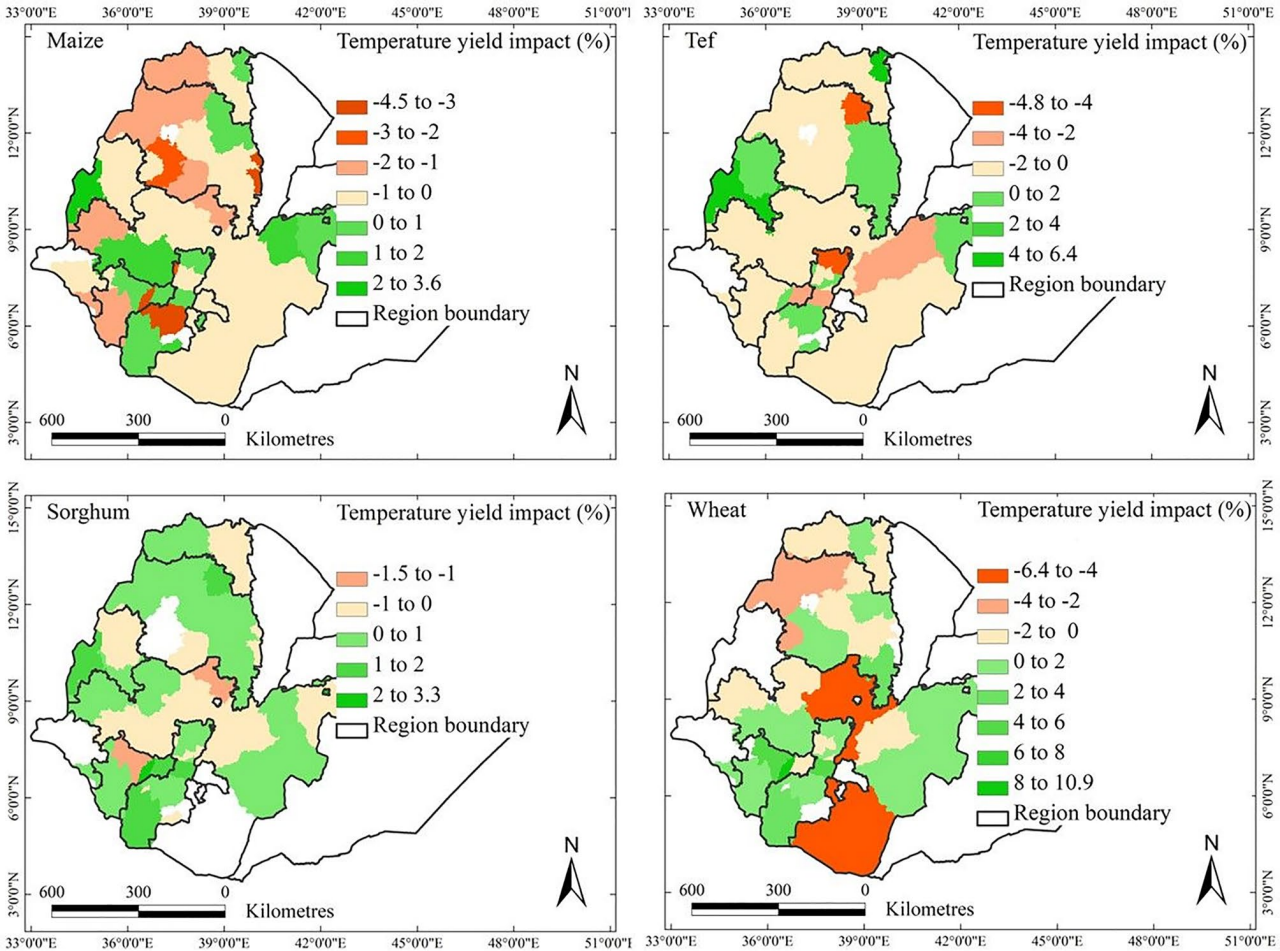
Patterns of the contribution of the trends in temperature at the level of administrative zone to the observed yields of maize, sorghum, tef, and wheat over 1995–2018 in the main crop-growing areas of Ethiopia. Percentages given are defined as crop yield trends due to trends in temperature divided by the overall trend in crop yield, all values taken per AZ over the period 1995–2018. White regions denote areas where a crop is not harvested or could not be examined.

**Fig. 7:**
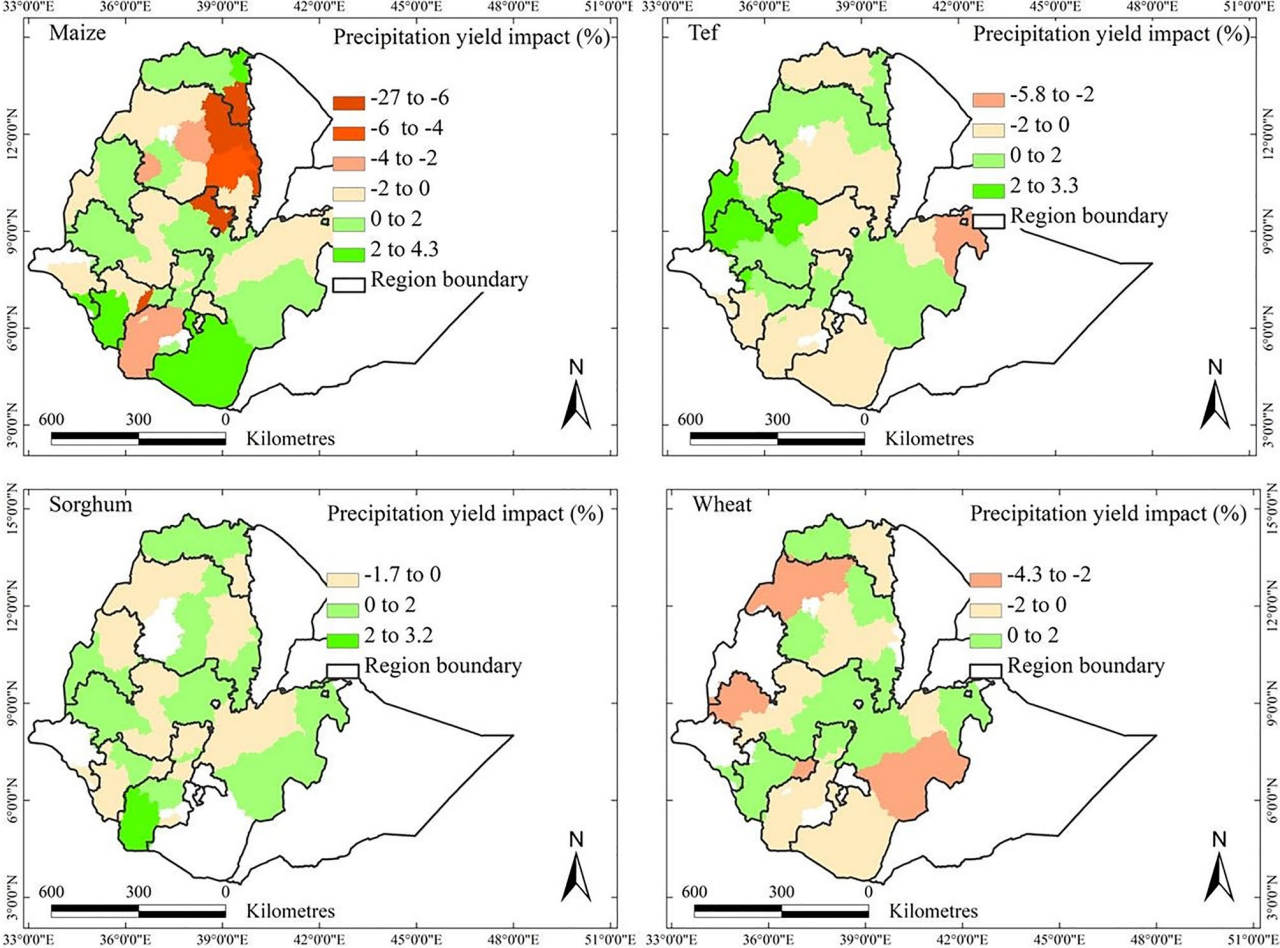
Contribution of the trends in precipitation at the level of administrative zone to the observed yield trends of maize, sorghum, tef, and wheat over 1995–2018 in the main crop-growing areas of Ethiopia. Percentages given are defined as crop yield trends due to trends in precipitation divided by the overall trend in crop yield, all values taken per AZ over the period 1995–2018. White zones denote areas where the crops of interest are not grown or where no data were available.

Table 3 summarises the model results at AZ level, with corresponding district level outputs available in Supplementary Table 4. The findings highlight that quadratic terms of temperature and precipitation have substantial impacts on crop yields. Crop yields were positively or negatively affected by the trends of temperature and precipitation (Figs. 6 and 7). The patterns differed for the four crops (Supplementary Fig. 7) and in almost all AZ impacts are rather small (well below 10%). For maize and tef, the number of AZs with temperature-induced losses was greater than those positively affected. Conversely, for sorghum and wheat, the number of AZs with temperature-induced gains surpassed those negatively affected.

**Table 3:** Output of applying a linear mixed-effects regression model at administrative zone level (Eq. 1). Response variable was crop yield (kg/ha), regressor variables were temperature, *T* (^o^C), precipitation, *P* (mm), and solar radiation, *R* (W/m^2^).

	Fixed effects			Random effects		Fixed effects			Random effects	
Variable	Coeff	Std. Error	*p*-value	Variance	Std. Dev	Coeff	Std. Error	*p*-value	Variance	Std. Dev
Maize						Tef				
Year	0.046	0.003	0.000	0.0003	0.017	0.046	0.003	0.000	0.109	0.012
T	− 0.002	0.010	0.472	0.0008	0.029	− 0.007	0.010	0.093	0.179	0.032
T^2^	− 0.012	0.006	0.030	0.0007	0.026	− 0.020	0.006	0.001	0.105	0.011
P	− 0.006	0.010	0.102	0.0007	0.026	0.007	0.010	0.097	0.178	0.032
P^2^	− 0.015	0.007	0.044	0.0002	0.005	0.005	0.008	0.035	0.183	0.034
R	− 0.019	0.010	0.047	0.0003	0.006	0.008	0.010	0.095	0.210	0.044
Residual				0.0350	0.188				0.453	0.205
Sorghum						Wheat				
Year	0.051	0.003	0.000	0.111	0.012	0.043	0.004	0.000	0.130	0.017
T	0.002	0.009	0.094	0.124	0.016	0.005	0.011	0.167	0.170	0.029
T^2^	− 0.008	0.006	0.014	0.093	0.009	− 0.024	0.007	0.001	0.161	0.026
P	0.007	0.010	0.062	0.198	0.039	− 0.007	0.009	0.016	0.161	0.029
P^2^	− 0.015	0.008	0.044	0.164	0.027	− 0.005	0.007	0.093	0.069	0.005
R	− 0.001	0.009	0.095	0.147	0.022	0.022	0.008	0.008	0.079	0.006
Residual				0.442	0.195				0.434	0.188

On average across the whole country, temperature-induced gains in yield in the AZs that were positively affected by 0.73, 0.81, 1.09, and 2.43% per year for maize, sorghum, tef, and wheat, respectively. Conversely, the temperature-induced losses in yield for these crops were 1.16, 0.33, 1.21, and 2.04% per year, respectively. Temperature-induced net impacts across the country were ‒0.43, 0.39, ‒0.11, and 0.30% per year for maize, sorghum, tef, and wheat, respectively.

Spatially, the trends in temperature had both negative and positive effects on crop yields, with the impacts differing by crop type and region. Maize yields were mostly negatively affected by temperature trends in the north-central and southern regions of the country, but in some areas the impacts were positive. In contrast, sorghum yield increased substantially across most regions, with yields increasing by up to 3.3% of the AZ average over the 24-year period. Tef yield was mainly negatively affected in the Tigray, Waghemra, West Gojjam, Gurage, and West Harerge AZs. Similarly, wheat yield was negatively affected in West Gojjam, Awi, East Shewa, and Borena.

The trends in precipitation predominantly had negative impacts in most AZs for maize and wheat crops. The precipitation-induced gains in yield between 1995 and 2018 were 1.3, 0.4, 0.6, and 0.7%, and the precipitation-induced losses in yield were 3.4, 0.4, 0.6, and 1.1%, for maize, sorghum, tef, and wheat, respectively. The net impacts for the country were generally more positive, with a decrease in yield of 2.1 and 0.4% for maize and wheat and increases of 0.04 and 0.02% for sorghum and tef, respectively.

Maize yields were affected by trends in precipitation most, with decreases up to 27% in the South Tigray, Waghemra, North Wollo, and North Shewa. The effect of trends in precipitation on Sorghum yield varied, with negative impacts in South Tigray, Gonder, Arsi, and Hadiya but positive impacts in most other regions. Tef yield was negatively affected by precipitation trends mainly in the central and southern parts of the country but positively affected in other AZs. Similarly, wheat yield was primarily negatively affected by precipitation trends in the western and southern administrative zones.

**Fig. 8:**
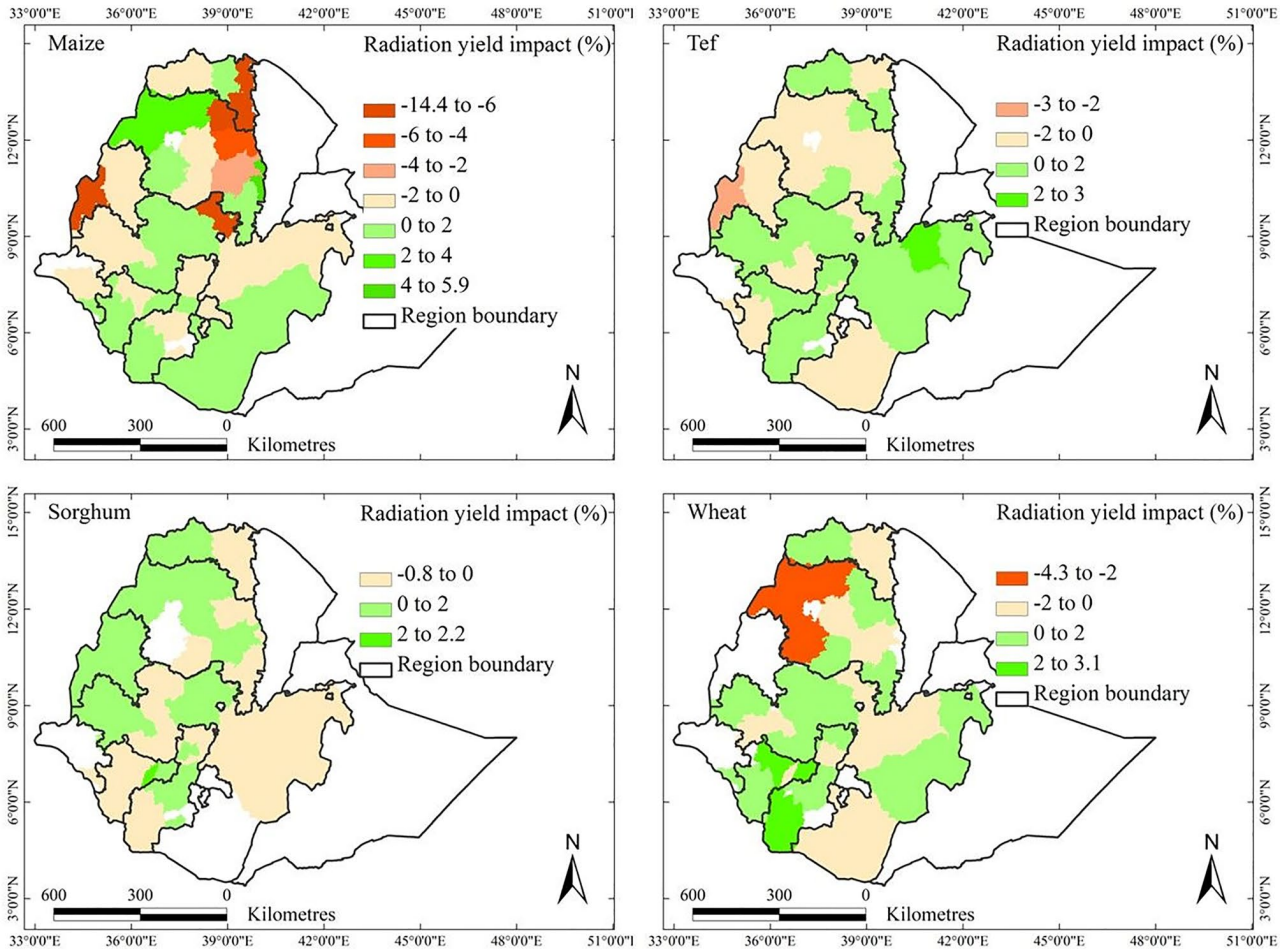
Contribution of the trends in solar radiation at the level of administrative zone to the observed yield changes of maize, sorghum, tef, and wheat over 1995–2018 in the main crop-growing areas of Ethiopia. Percentages given are defined as crop yield trends due to trends in solar radiation divided by the overall trend in crop yield, all values taken per AZ over the period 1995–2018. White zones denote areas where the crops of interest are not grown or where no data were available.

The yield of all crops benefitted from the trends in solar radiation in most parts of the country, but the impact was small (Fig. 8). The trends in solar radiation positively affected yields on average by 0.88, 0.37, 0.53, and 0.97% per year for maize, sorghum, tef, and wheat, respectively, over the entire period 1995–2018. The negative impacts (up to ‒14.4% per year) on maize yields happened mainly in Eastern Tigray, Wollo, Waghemra, Asosa, and North Shewa AZs, but all other areas were positively affected. Sorghum yield, however, decreased by up to 0.8% per year in the Harari, Arsi, East Harerge, Sheka, and South Omo AZs. Tef yield was negatively affected (up to ‒3% per year) in West Gojjam, Asosa, Metekel, North Shewa, South Wollo, Central Tigray, and Borena. Wheat yield was negatively affected (up to ‒4.3% per year) in the Awi, Gondar, West Gojjam, Eastern Tigray, North Shewa, East Shewa, and Borena AZs.

#### Dominant climatic drivers of yield trends

**Fig. 9:**
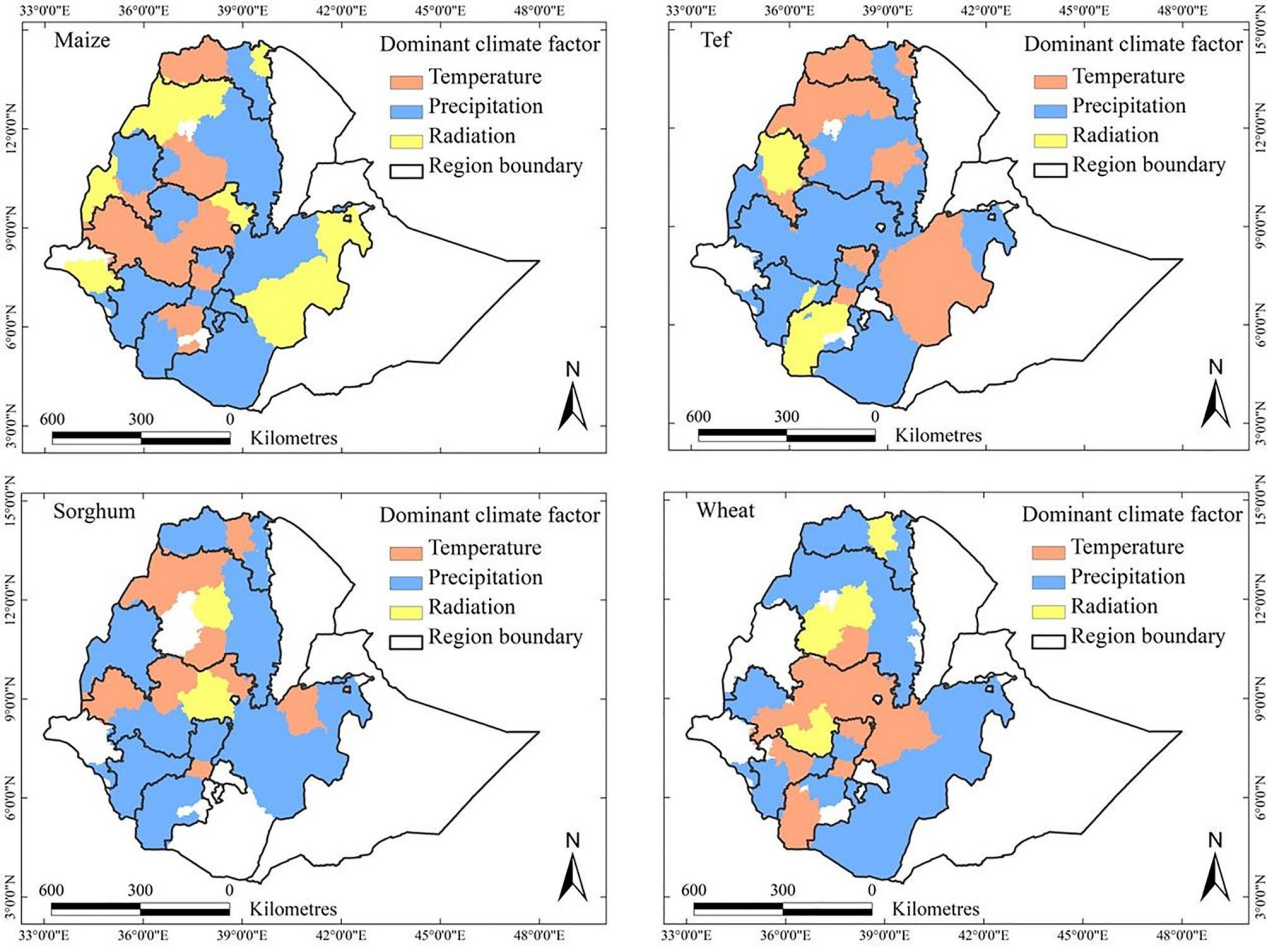
Individual climatic factors dominating the climate-driven trends in maize, sorghum, tef, and wheat yields, respectively, between 1995 and 2018 in Ethiopia. White zones denote areas where the crops of interest are not grown or where no data were available.

To identify the dominant climatic driver for each crop and region, we assessed which of the three variables, temperature, precipitation, or radiation, contributed most to the observed yield trends (Fig. 9). Temperature was the dominant climatic factor in 28.5% of the maize-growing areas, which accounted for 46.8% of the national maize production, particularly located in the western and southeastern regions. Sorghum yield trends were influenced by temperature in 25.6% of the growing areas, contributing to 19.4% of the national sorghum production. For tef, temperature was the dominant factor in 34.3% of the tef-growing areas, particularly in the northern and southern areas. Temperature was the dominant climate factor for wheat yield trends in some central parts of the country, covering 27.7% of the total (national) wheat-growing area.

Precipitation was a significant climatic factor influencing the trends in crop yields across various regions. For maize, precipitation was the dominant factor in 42.1% of the maize-growing AZs, contributing about 35.9% to the national maize production. Sorghum yield in the majority of the crop-growing areas was primarily influenced by precipitation, which was the dominant factor in 68.2% of the growing areas, equivalent to 76.1% of national sorghum production. Similarly, precipitation was the dominant factor for tef yield in 56.3% of the tef-growing areas, making it the dominant driver for most of the crop-growing areas. For wheat, precipitation was the dominant factor affecting yield trends in most parts of the country, accounting for 67% of the total wheat-growing AZs.

Solar radiation also influenced crop yield trends but was less than temperature and precipitation. For maize, radiation was the dominant factor in 29.4% of the maize-growing areas, contributing 17.4% to the national production. Sorghum yield trends were influenced by radiation in a few areas, although its role was less pronounced that of as precipitation and temperature. For tef, solar radiation was the primary factor in 9.4% of the production areas. Wheat yield trends were dominantly controlled by solar radiation in Central Tigray, South Gonder, West Gojjam, Awi, and Jimma, covering 10.3% of the total wheat-growing areas. The substantial spatial variation in the dominant climatic factors clearly demonstrated that the impacts of climate were local or regional and should be addressed by considering local environmental settings.

#### Climate’s contribution to overall yield trends

**Fig. 10:**
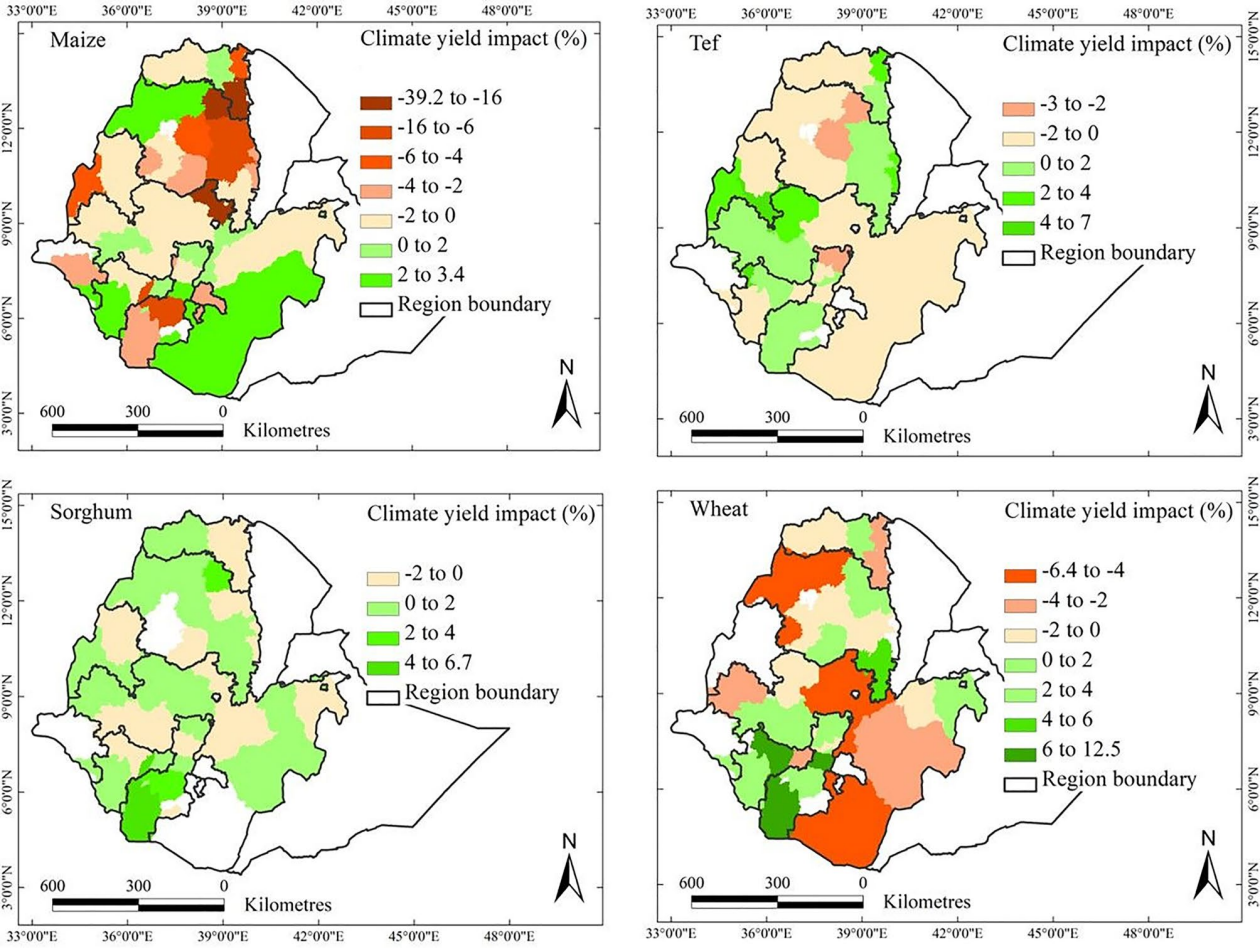
Contribution of all climatic factors to the trends in maize, sorghum, tef, and wheat yields in the main crop-growing regions of Ethiopia. Percentages given are defined as crop yield trends due to trends in climatic factors divided by the overall trend in crop yield, all values taken per AZ over the period 1995–2018. White zones denote areas where the crops of interest are not grown or where no data were available.

We further assessed the overall contribution of climatic trends by comparing climate-induced yield changes with total observed yield trends. This approach isolates the climatic signal from other influences such as technology advancements. Figure 10 illustrates the overall impact of all climatic factors (temperature, precipitation, solar radiation) on the total trend in yield for 1995‒2018. The climate impact varies from − 39.2% (maize, Waghemra AZ) to 12.5% (wheat, Segen people AZ). Specifically, the contribution of climatic trends to the trends in maize yield varied from  − 39.2 to + 3.4% in main production areas such as Shewa, Gojjam, Wollo, South Tigray, and East Welega. However, the average effect on crop yields was small, because gains in some regions offset losses in others.

On average at the country level, the climatic trends contributed to ‒3.2% for maize, 0.6% for sorghum, 0.2% for tef, and 0.7% for wheat, indicating that other factors were driving the changes in crop yield (see also Supplementary Fig. 7). The impacts were positive in most regions of the country, despite the average net decrease of 3.2% per year for maize. These findings also highlight that the effects of climatic trends on crop yields are complex and vary with location and crop type.

We also evaluated the effects of climatic trends derived from selected productive districts’ mixed-effects models in comparison with those obtained from the corresponding AZ mixed-effects models. Our analysis found a substantial similarity in the outcomes across these spatial levels (see Supplementary Fig. 8), indicating consistency in the climatic signal captured at both district and zonal scales.

### Comparison of outputs from linear mixed-effects and first-difference regression models

To assess the robustness and consistency of climate-yield relationships across different temporal and statistical frameworks, we compared the estimated impacts of climate variables on crop yields derived from the linear mixed-effects model (LMM) and the first-difference regression model (FDRM) (see Supplementary Fig. 9). The LMM quantifies the long-term contributions of both climatic and non-climatic factors to crop yield trends, incorporating spatial heterogeneity through random effects at the AZ level. In contrast, the FDRM isolates short-term, interannual yield responses to year-to-year climate variability by removing temporal trends and focusing on within-AZ annual fluctuations.

The average overall climatic impact estimates obtained using the first-difference regression model were ‒ 0.59% per year for maize, 0.53% per year for sorghum, 0.26% per year for tef, and 0.68% per year for wheat over 1995‒2018. When compared to the LMM outputs, the strength of the association between the climatic impact estimates from the two models varied across crops, as indicated by the CD values, 0.61 for maize, 0.46 for sorghum, 0.52 for tef, and 0.63 for wheat. These values suggest a moderate to strong correlation between the LMM and FDRM models, with wheat and maize exhibiting the highest agreement, while sorghum displayed the weakest association.

Notably, sorghum exhibited the lowest CD value (0.46), particularly in North Shewa and Oromia Special AZs, where the first-difference model estimated positive yield impacts, whereas the mixed-effects model indicated negative effects. This discrepancy highlights potential regional variations in climate responsiveness or the influence of additional factors not captured equally by both models. Similarly, in East Tigray and Konta Special AZs, both models estimated negative impacts of climate trends on yield, but the magnitude of these effects differed.

Overall, while both models produced qualitatively similar results, they differed in the magnitude and, in some cases, the direction of estimated impacts in specific regions. These differences may be attributed to a combination of limited sample sizes and inherent structural differences between the models. The linear mixed-effects model accounts for hierarchical structures through random effects, whereas the first-difference regression model removes time-invariant effects but does not effectively handle group-level heterogeneity. By comparing them, we increase confidence in robust signals (e.g., precipitation effects on sorghum and wheat) and identify areas where model assumptions affect interpretation.

### Contribution of non-climatic factors to crops yield variability

To disentangle the influence of non-climatic factors, particularly technological advancements, from climate-induced yield trends and variability, we incorporated a temporal trend (*year*) in the mixed-effects regression model. This variable served as a proxy for gradual, systematic improvements unrelated to climate, such as improvements in agronomic practices, increased input use, improved irrigation systems, and the development of high yielding cultivars.

The fixed effect of the year variable was positive and highly significant (*p* < 0.001) across all four cereal crops (Table 3), indicating a consistent upward trend in yields due to non-climatic influences for 1995–2018 period. Maize and tef both show an estimated year coefficient of 0.046, implying an average increase of 4.6% per year in yield, independent of climatic variables. Sorghum shows the highest year effect (0.051), pointing to relatively greater benefits from non-climatic advancements in sorghum-growing AZs. Wheat follows closely with a year coefficient of 0.043, reflecting significant technological progress in wheat growing areas.

**Fig. 11:**
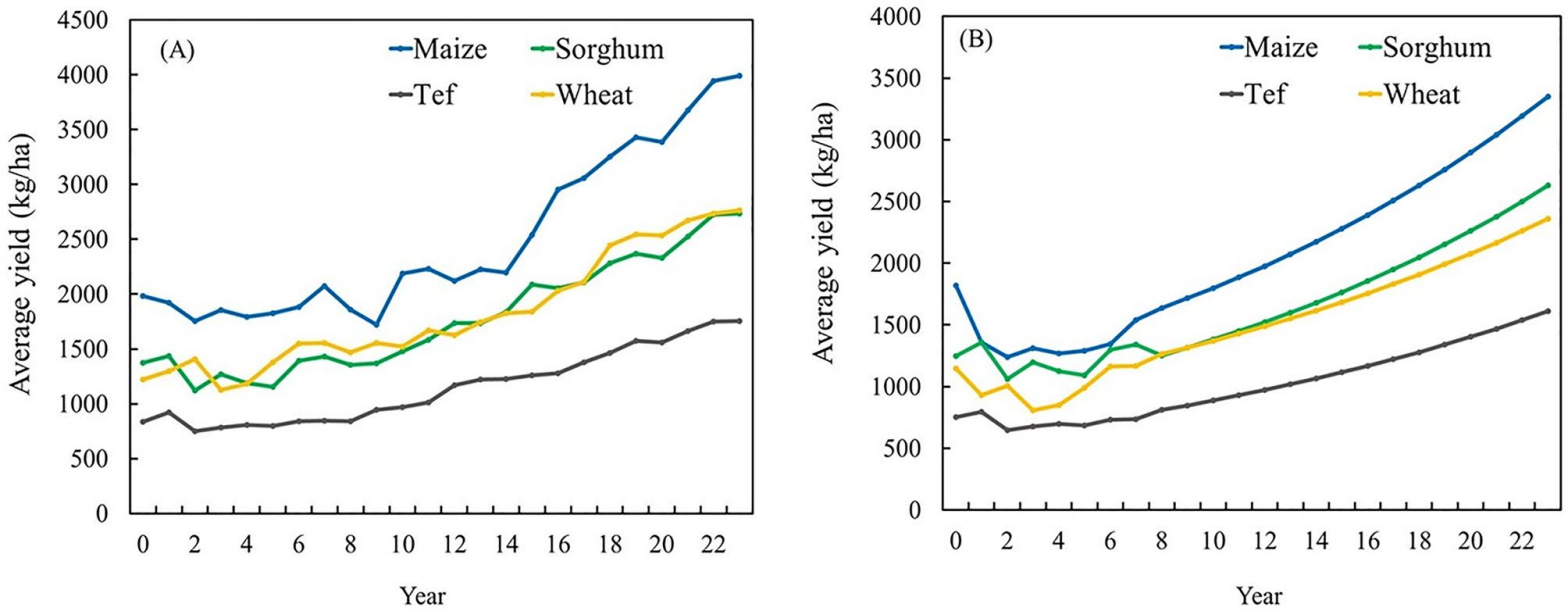
Average annual yield for maize, sorghum, tef, and wheat across all administrative zones for the period 1995–2018 (**A**). Partial dependence plot for time (**B**). Maize, sorghum, tef, and wheat are denoted by blue, green, black, and orange lines, respectively. Partial dependence plots graph the independent variable against the model outcome, after accounting for the average effect of other independent variables in the model.

These temporal trends are further visualized in the partial dependence plots for time (Fig. 11). Figure 11A illustrates a clear upward trend in the average annual yields of maize, sorghum, wheat, and tef across the AZs for the study period. Notably, maize exhibits the highest yield gains over time, followed by wheat and sorghum, while tef shows relatively modest improvement. These trends are further substantiated by the partial dependence plots in Fig. 11B, which isolate the marginal effect of time on crop yield after accounting for other covariates in the model. The consistent positive slope in these plots for all crops indicates a strong temporal component contributing to yield gains, interpreted here as a proxy for non-climatic factors, primarily technological advancements. Our estimates indicate that in the period 1995–2018 the temporal advancements contributed to country level yield increase of approximately 96.8 kg/ha per year for maize, 71.1 kg/ha per year for sorghum, 70.5 kg/ha per year for tef, and 44.9 kg/ha per year for wheat.

Random effects associated with the year variable indicate substantial variability in yield trends across AZs (Table 3). For instance, the standard deviation of the random intercept for year in tef (0.012) is notably smaller than for sorghum and wheat (both 0.017), suggesting more homogeneous yield trends in tef compared to other crops. The high residual variances for sorghum and wheat (0.442 and 0.434, respectively) reflect substantial unexplained heterogeneity.

## Discussion

Understanding the spatial and temporal variability and trends of climatic and non-climatic factors, and their impact on crop yields, is critical for devising effective adaptation strategies, especially in regions like Ethiopia where agriculture is highly climate-sensitive. Our findings support the growing body of evidence that climate change, particularly warming, is among the major challenges affecting agricultural production in the country.

Our analysis showed significant positive trends in temperature across most crop-growing areas, consistent with prior studies reporting national and regional warming over recent decades (e.g., NMA^28^, Tesfaye et al.^29^, and Gebrehiwot and van der Veen^56^). For instance, we found that growing season temperature increased by up to 0.83 °C per decade in some areas. These findings align with global trends documented by the IPCC^57^ and reinforce the narrative of progressive warming in the region. Precipitation patterns, in contrast, exhibited high interannual variability with no consistent trends across regions. While some areas experienced significant declines, others showed no discernible change or even slight increases. This variability reflects the complexity and region-specific nature of precipitation trends in Ethiopia, as also reported by Tekleab et al.^58^, Seleshi and Camberlin^59^, and Conway^60^.

Despite the observed climatic variability, crop yields for maize, sorghum, tef, and wheat generally increased across most AZs during 1995–2018. Yield trends ranged from 60.6 kg/ha/year for tef to 154.4 kg/ha/year for maize. Our linear mixed-effects model estimated that climate-induced yield changes accounted for up to 39.2% (maize, Waghemra AZ) of the overall yield trend. On average at the country level, climatic trends contributed positively to yield trends for sorghum (0.6%), tef (0.2%), and wheat (0.7%) but were associated with a decline in maize yields by ‒3.2%. Similar results with positive contributions of trends in climate factors were reported by Hadgu et al.^27^ and Asseng et al.^61^. These studies suggested that an increase in the availability of water was the primary factor driving the higher crop yields. Ginbo^62^ and Yang et al.^12^ observed positive effects of climatic trends on maize and tef yields in Ethiopia, respectively. Ray et al.^63^ also documented an increase in sorghum yield by 0.7% for the period 1974–2008 across Sub-Saharan Africa, which they attributed to recent climate changes. In contrast, other studies^4,64^ presented results that differed from our findings, highlighting the adverse impacts of climatic trends. Some previous reports have also noted mixed results for various crop yields^62,65^. The above finding differences are probably due to differences in the study areas, methods, time periods.

Other studies have also documented significant relationships between climatic factors, such as seasonal temperature and precipitation, and crop-yield trends and variability in Ethiopia^27,39,40,42,66^ and elsewhere^4,35,36,64^. Ray et al.^32^ and Lobell et al.^4^ demonstrated the intricacy of the mechanisms and factors affecting crop yields, making it difficult to ascribe changes in yields to a particular set of factors.

Long-term climate-induced yield trends generally align with the impacts of interannual climate variability on crop yields, though they differ in magnitude (Tables 2 and 3). Wheat shows the highest sensitivity to temperature across both time scales, with positive responses to short-term variability and long-term warming, consistent with prior findings on its adaptability in cooler highland areas^4,32^. In contrast, maize shows lower sensitivity to interannual temperature variability despite significant negative effects from long-term temperature trends, reflecting its vulnerability to cumulative heat stress during reproductive stages^12,55^. Tef demonstrates positive sensitivity to temperature variability but a slight negative response to temperature trends. Precipitation trends also emphasize these distinctions. Maize and wheat are more adversely affected by long-term precipitation declines than by interannual variability. Conversely, sorghum and tef benefit from increased rainfall over both time scales, reflecting their adaptability to semi-arid conditions and efficient water use^27,61^. Solar radiation further illustrates these complexities. Maize is negatively affected by both short- and long-term increases in radiation, likely due to evapotranspiration stress. While tef benefits from enhanced radiation, possibly due to its better ability to convert solar energy into yield under suitable moisture conditions^4^.

These observed patterns emphasize the importance of considering both long-term climatic trends and short-term variability in crop-climate analyses. Crops like maize, which suffer significant losses under persistent climate stress and are relatively insensitive to year-to-year variability, are particularly vulnerable. In contrast, more resilient crops such as sorghum and tef, which tolerate both trends and variability, are promising alternatives for regions facing increasing climate uncertainty.

Maize’s heightened sensitivity to climate stress is primarily due to its physiological traits^67^. Its rapid growth and large leaf area lead to high water demand driven by elevated transpiration^10^. Although maize benefits from the efficient C4 photosynthetic pathway, it still requires substantial moisture, especially during flowering and grain filling, to sustain nutrient transport and turgor pressure^36^. Its relatively shallow root system limits access to deeper soil moisture, making it highly vulnerable to short-term drought^68^. Moreover, its reproductive stages, particularly flowering, are extremely sensitive to heat stress, where elevated temperatures can cause pollen sterility and poor fertilization, reducing kernel set and yields^69^. These stages have minimal recovery potential, compounding maize’s vulnerability under Ethiopia’s projected climate extremes^57,66^.

The variability and trends of crop yield are affected by a multitude of factors, both climatic and non-climatic^4,16,32^. Climate is lately often considered the primary driver of variability and trends in yield, but non-climatic factors determine most of the crop productivity. Our results indicated that the relative contribution of trends in climate factors to the trends in crop yield for the main crop-growing areas was small (Fig. 10), highlighting the dominant influence of non-climatic factors. Our analysis using the partial dependence plots for time revealed that crop yields significantly increased over time (Fig. 11). The fixed effects estimates for year in Table 3 further support this conclusion. The fixed-effect coefficients for the year variable are highly significant (*p* < 0.001) and positive across all four crops, ranging from 4.3% per year (wheat) to 5.1% per year (sorghum), indicating a consistent, positive time trend in yields that cannot be explained by climate alone. Additionally, the random effects variance for the year term across AZs suggests heterogeneity in the rate of technological advancement among AZs, possibly reflecting differences in access to extension services, infrastructure, input markets, or development programs. For example, AZs with better access to hybrid seeds, fertilizers, irrigation infrastructure and climate-smart agricultural practices likely experienced faster yield gains over time, while marginal or remote AZs may have benefited less.

Nevertheless, it is worth acknowledging that the temporal trend may also capture minor effects from other long-term processes such as rising atmospheric CO_2_ concentrations. However, as noted in Lobell et al.^4^ and McGrath and Lobell^55^, the yield benefit from CO_2_ fertilization is generally modest compared to the impact of direct technological interventions. Moreover, the differences in the rate of yield gain among AZs suggest that technological progress was more influential. Therefore, we interpret the observed temporal yield gains as being primarily driven by non-climatic factors, with a minor and indirect contribution from environmental changes such as CO_2_ enrichment.

It is well acknowledged that factors such as land management, irrigation, improved seed varieties, and crop diversity play pivotal roles in determining the development and productivity of crops in Ethiopia^19,70–72^. For example, soil degradation and drought in northern Ethiopia increased annually prior to the 1980s^29,73–75^. The Ethiopian government, however, subsequently initiated policies in the north focusing on land-rehabilitation programmes. These interventions increased crop production by the widespread adoption of practices for conserving soil and water^42,76,77^ and the expansion of irrigated land facilitated by water-harvesting techniques^78,79^.

Climate plays an important role in influencing crop yield variability and trends. However, non-climatic factors, particularly technological advancements, have had a much larger impact on overall crop yields over the 24-year period. These advancements not only enhance yield levels but also influence the variability and stability of crop production over time. The relatively low impact of climate on yields may be attributed to the minimized sensitivity of low-yielding or stressed crops (Table 2) to weather variations^9,80^. It is therefore essential for policymakers, experts and local communities to consider both climatic and non-climatic factors to optimize crop yields and strengthen food security in Ethiopia and the broader region.

This study quantitatively analysed to what extent climatic variability and its trends affected crop productivity in the main crop-growing areas of Ethiopia for 1995–2018. Our study primarily relied on validated data from existing sources, but uncertainties in the accuracy of the data could have influenced the analysis. For example, the meteorological data obtained from the Ethiopian National Meteorological Agency (NMA) were only available from a limited number of stations within or near the study area (Fig. 1), particularly in lowland regions of the country. This limitation may have led to uncertainty in the interpolated product and subsequent regression analyses. Numerous studies e.g.^12,32,42,81,82^ have indicated that inadequate and insufficient data pose large obstacles in developing countries such as Ethiopia.

Additionally, the solar radiation data used in this study were derived from the DOE-II reanalysis dataset, with an original spatial resolution of 0.5°, and subsequently downscaled to 1 km using bilinear interpolation. As reanalysis-based modeled products, these data introduce additional uncertainties, particularly in regions with complex terrain or high climatic variability. Although our findings showed that solar radiation trends had a smaller and more spatially heterogeneous impact on crop yield trends than temperature and precipitation (Fig. 8), uncertainties in the radiation data may contribute to residual variance in the models.

Our modeling approach also assumes linearity and additivity in fixed and random effects. Although we included quadratic terms to capture some nonlinearities, other interactions or threshold effects may not have been fully captured. Temporal autocorrelation was modeled using an AR(1) structure, but potential spatial autocorrelation between neighboring AZs was not addressed.

Importantly, while our model included nonlinear climate-yield relationships via quadratic terms, it did not explicitly assess discrete extreme events (e.g., dry spells, heatwaves, flash floods). Such events, often responsible for severe interannual crop losses, occur independently of seasonal averages and require specific indices (e.g., Standardized Precipitation Evapotranspiration Index, heat stress duration) for detection. The lack of high-resolution, sub-seasonal climate data constrained our ability to include these metrics, representing a significant limitation. Future research should prioritize the integration of extreme climate indices to better understand compound and sequential climate shocks under changing conditions.

We also recognize that factors such as wind speed, heat stress, atmospheric CO_2_ concentration, soil moisture and localized agronomic practices, although partially accounted for through AZ level random effects, are not directly included in the model due to data limitations. This omission may result in residual confounding. Similarly, while the year variable proxies for technological advancement, it may also reflect unmeasured socio-economic or policy changes that can influence yield trends.

Our study employed a combination of linear mixed-effects models and first-difference regression to assess both long-term trends and short-term interannual variability in crop yields. The linear mixed-effects model framework effectively accounted for the hierarchical data structure and allowed us to disentangle climatic and non-climatic influences, which is especially valuable in data-scarce contexts where technological change is not directly observed. First-difference regression complemented this by capturing yield sensitivity to year-to-year changes in climate variables. This dual approach improved the robustness and consistency of our findings across statistical perspectives. However, our methodology does not establish causal relationships. Unlike causal inference frameworks, our models assume climate variables are exogenous and do not control for unobserved, time-varying confounders. The absence of exogenous shocks or intervention data precluded the applicability of techniques such as difference-in-differences^83^. Future research should explore causal methods where data permit, particularly in studies of targeted agricultural interventions.

Our findings nevertheless have important implications for understanding the effects of climatic and other driving factors on crop yield variability and trends in Ethiopia, where agriculture is predominantly smallholder-based. They provide valuable evidence for understanding recent progress in crop yields and for informing sustainable adaptation and mitigation strategies in the context of climate variability and human-induced changes. In Ethiopia, crop production is predominantly rainfed, with < 1% of the land irrigated. When livelihoods depend on rain, areas that do not receive adequate rainfall in some years may face food insecurity, even if national food production remains unaffected^13,84^. Minor climatic shocks in these areas can lead to crop failure, affect farming choices and practices, cause shortages in water and pasture, decrease livestock production and market value, and ultimately contribute to food insecurity^39,85^.

Current research suggests that the effects of climate change and extreme weather will persist and have increasingly severe consequences on agricultural productivity in the country^10,27,42^. This situation is expected to decrease the adaptive capacity of farmers, intensify poverty, and widen the food gap. The country must address these challenges by adopting crop- and location-specific policies and strategies that encourage resilient agricultural practices capable of withstanding the impacts of climatic variability and change, including enhancing the management of water resources and fostering the cultivation of crop varieties resilient to droughts in regions facing climatic challenges. Addressing yield gaps in climatically suitable areas is also vital to augment national food availability.

We recognise the significant role that technological advancements have played in enhancing crop production across the country. Innovations, including conservation agriculture, rainwater harvesting, and improved crop genotypes, have significantly contributed to increased crop production in drought-prone areas^86,87^. However, the long-term effects of these interventions on yield stability and ecosystem services remain unclear due to limited long-term field data^4^. Using a mixed-effects regression model, this study quantifies the relative contributions of climatic and non-climatic factors to crop yield variability and trends. Our spatial and temporal mapping of climate and yield data provides insights into how these drivers interact under water-limited conditions. These findings support the evaluation of current adaptation and mitigation strategies and offer guidance for improving agricultural resilience under increasing climate variability. Understanding this interplay is crucial for sustaining productivity in dryland systems and developing robust responses to future environmental challenges.

## Conclusions

We investigated how variations and trends in mean air temperature, precipitation, solar radiation and non-climatic factors such as technology improvement affected the variabilities of maize, sorghum, tef, and wheat yields at AZ and district levels in Ethiopia during the main growing season over the period 1995–2018. Our findings identified a notable disparity in the influence of climatic variability and trends across different crop-growing areas. The effects were not significant in some areas, but over 50% of the interannual variability in yield was attributed to climatic factors in most parts of the crop-growing areas. However, the contribution of long-term climatic trends to yield changes was limited at the national level, averaging only − 3.2% for maize, + 0.6% for sorghum, + 0.2% for tef, and + 0.7% for wheat. Nevertheless, localized adverse impacts were considerable, with climate-induced yield losses reaching up to − 39.2% for maize in single AZs. Precipitation trends were notably the primary climatic determinants in up to 68% of the main crop-growing areas, contributing to approximately 76% of the total crop production in the country.

Overall, the findings highlight the complex interplay between climatic and non-climatic influences on crop productivity. While climate variability and trends, particularly in precipitation and temperature, have had measurable effects on crop yields, non-climatic factors have played a more decisive role. Technological improvements, including enhanced crop varieties, better agronomic practices, and increased input use, contributed to substantial and consistent yield gains, 4.6% per year for maize, 5.1% for sorghum, 4.6% for tef, and 4.3% for wheat. These insights underscore the critical need for crop- and location-specific strategies that enhance adaptive capacity by addressing both climatic risks and leveraging non-climatic innovations to improve agricultural resilience and ensure food security in the region.

## Supplementary Information

Supplementary Information.

## Data Availability

The datasets used and analyzed during the current study are available from the corresponding author upon reasonable request.
