# Supplementary material for: Spatiotemporal climatic signals in cereal yield variability and trends in Ethiopia
Source: Sci Rep. 2025 Oct 23;15:37159. doi: 10.1038/s41598-025-23452-7 (PMC12549832; doi:10.1038/s41598-025-23452-7)
Supplement: Supplementary file 1 — Supplementary Information. [file 41598_2025_23452_MOESM1_ESM.pdf]

Supplementary information for

Spatiotemporal climatic signals in cereal yield variability and trends in Ethiopia

[1,2,*]Kidist Abera, [1]Sebastian Gayler, [3]Hans-Peter Piepho, [1]Thilo Streck

[1]Institute of Soil Science and Land Evaluation, Biogeophysics, University of Hohenheim, Stuttgart, Germany

[2]Ethiopian Institute of Agricultural Research, P.O. Box 2003, Addis Ababa, Ethiopia

[3]Biostatistics Unit, Institute of Crop Science, University of Hohenheim, Stuttgart, Germany

Introduction

This supplementary information contains figures and tables that support our findings and statistical analyses, illustrating how the spatiotemporal variations and trends in mean air temperature, precipitation, solar radiation and non-climatic factors such as technology improvement affected the variabilities of maize, sorghum, tef, and wheat yields. The data is presented at both administrative zone and district levels across Ethiopia's major crop-growing regions for the period 1995–2018.

List of Supplementary Figures, Tables and methods details

I. Supplementary Figures

Fig. 1. Maps of regional and administrative zonal boundaries of Ethiopia.

Fig. 2. Spatial distribution of growing season (June–September) average values of climate variables from 1995−2018.

Fig. 3. Crops (maize, sorghum, tef, and wheat) yield and climate (temperature, precipitation, and radiation) anomalies over the major growing areas of Ethiopia for the period 1995−2018.

Fig. 4. Time series (1995–2018) of growing season mean temperature and total precipitation for five representative meteorological stations in the west, east, and central parts of Ethiopia.

Fig. 5. Area coverage and average yields (1995–2018) of maize (A), sorghum (B), tef (C), and wheat (D) in the administrative zones of the major crop growing areas of Ethiopia.

Fig. 6. Absolute changes in crop yields (kg/ha) for maize (A), sorghum (B), tef (C), and wheat (D) in the major crop growing areas of Ethiopia (1995–2018).

Fig. 7. Climate-induced impacts on crop yields (maize, sorghum, tef, and wheat) in the major crop growing areas of Ethiopia (1995–2018).

Fig. 8. Estimated net effects of climatic trends on crop yield trends across primary producer districts and their respective administrative zones.

Fig. 9. Relationship between yield impacts estimated using mixed-effects regression models and first-difference regression models.

Fig. 10. Scatter plots showing residuals versus predicted yield for observed (A–D) and transformed (E–H) data across different crops: maize (A, E), sorghum (B, F), tef (C, G), and wheat (D, H).

Fig. 11. Comparison of different model specifications using separately modeled temperature variables: mean temperature (TMEAN), maximum temperature (TMAX), and minimum temperature (TMIN).

II. Supplementary Tables

Table 1. Meteorological data and trends for growing season (June–September) average air temperature and total precipitation (1995–2018).

Table 2. Trends and variation of yield for maize, sorghum, tef, and wheat in productive districts of Ethiopia (1995–2018).

Table 3. Meteorological data and trends for growing season (June–September) average air temperature and total precipitation (1995–2018) across administrative zones of Ethiopia.

Table 4. Effects of fixed and random variables on maize, sorghum, tef, and wheat yields at selected district levels.

Table 5. Comparison and validation of monthly simulated climate datasets with the observed dataset during 2010−2014.

III. Supplementary Methods

A. CSA sampling methodology

B. Development of administrative zonal- and district-based crop yield and climate data

C. Gridding and validation of climate data

D. Trends and covariation estimation

E. First difference regression model

F. Mixed-effects model implementation

I. Supplementary Figures

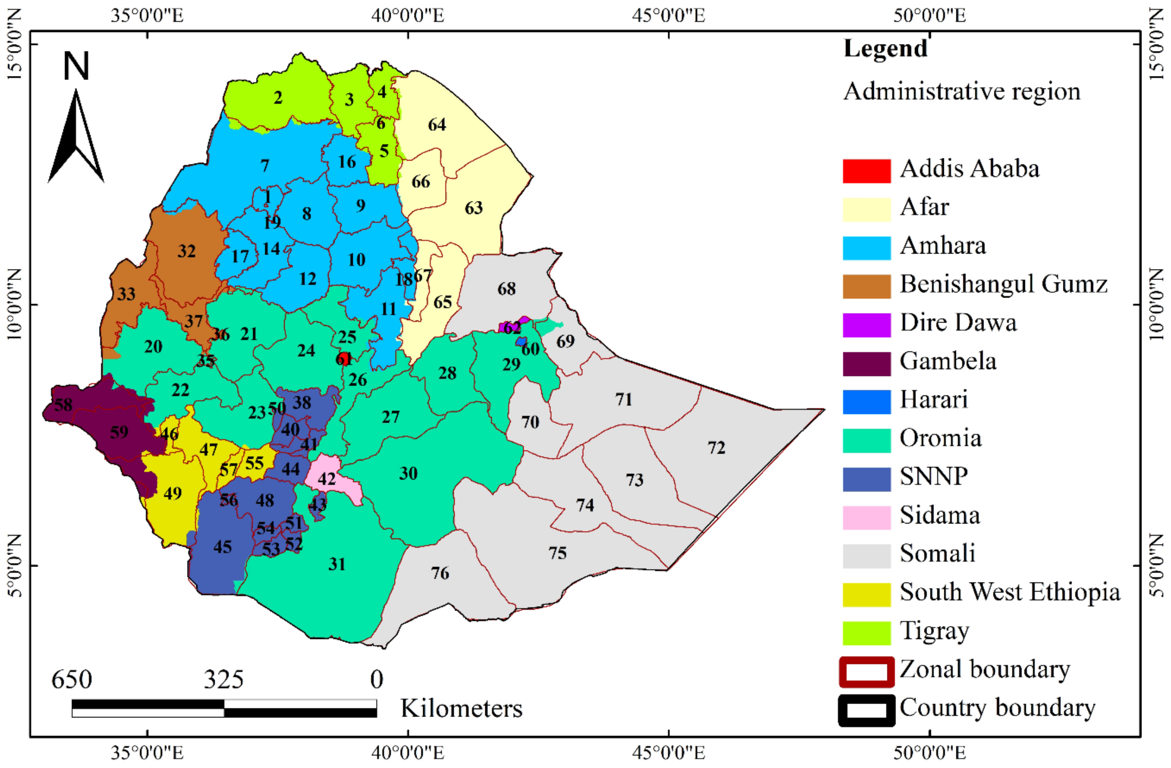

Fig. 1. Maps of regional and administrative zonal boundary of Ethiopia. Administrative regions are presented in different colors. The administrative zones are identified by numbers. The numbers and names of the administrative zones are provided in Appendix B, Table B.3.

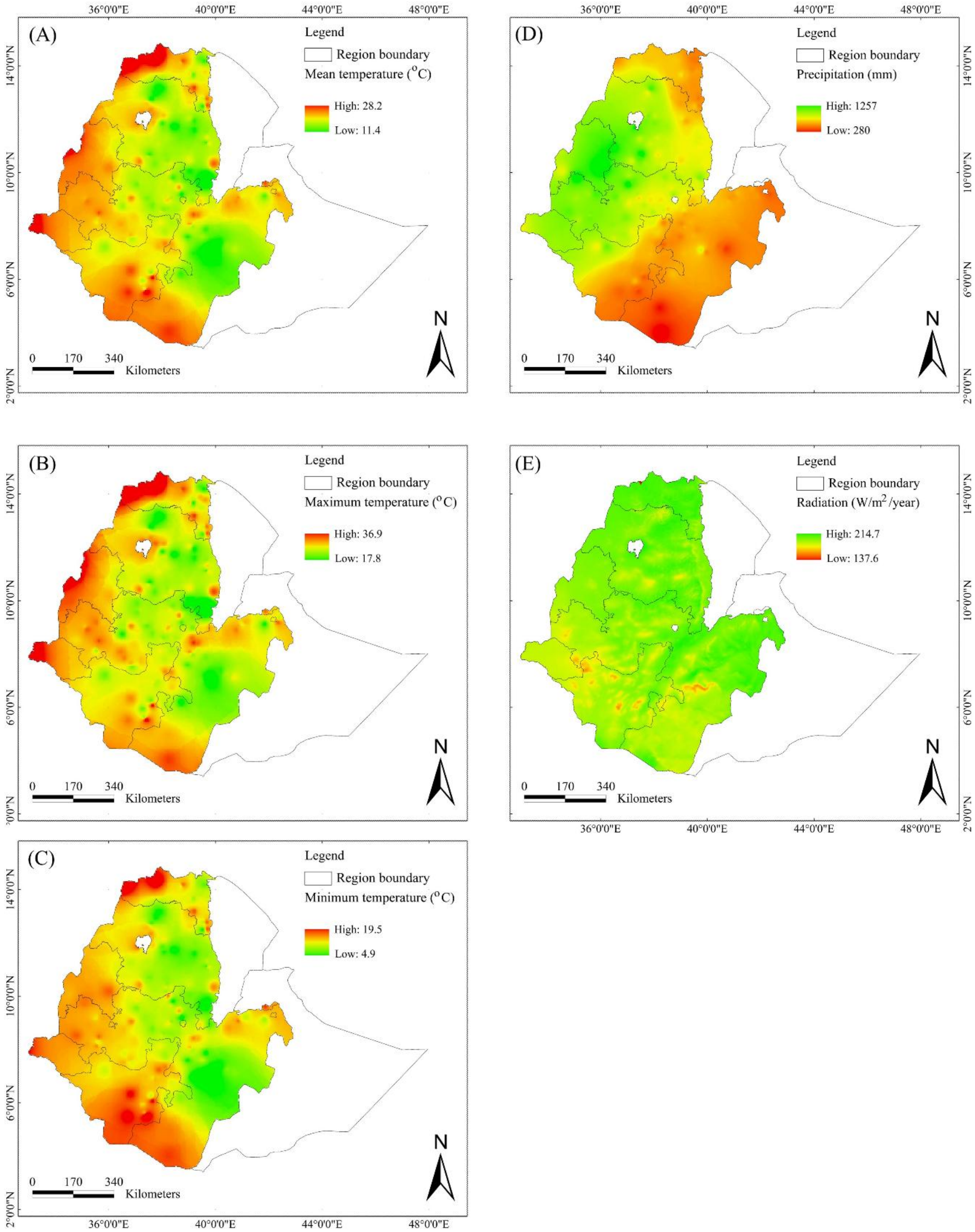

Fig. 2. Spatial distribution of growing season (June–September) average values of climate variables from 1995−2018. Sub-figures (A), (B), (C), (D) and (E) are spatial distributions of mean, maximum and minimum temperatures, precipitation, and solar radiation, respectively.

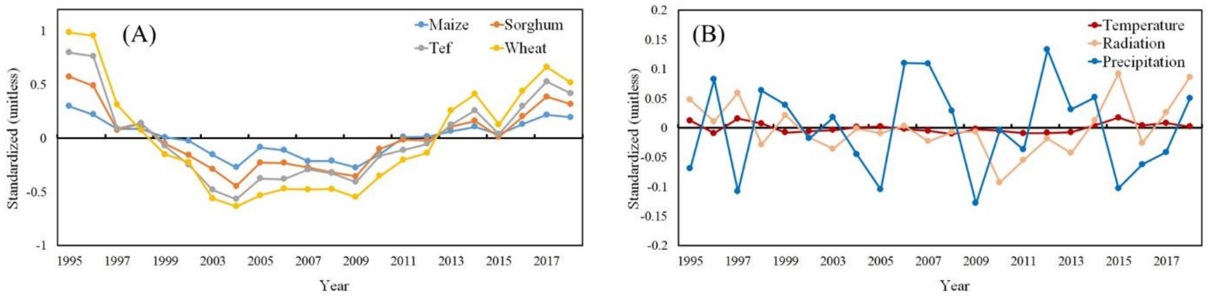

Fig. 3. Crops (maize, sorghum, tef and wheat) yield and climate (temperature, precipitation and solar radiation) anomalies over the major growing areas of Ethiopia for the period 1995−2018. The changes were calculated by subtracting the mean value of the period 1995−2018 from the annual growing season value for each year. Subsequently, the resulting value was divided by the mean value.

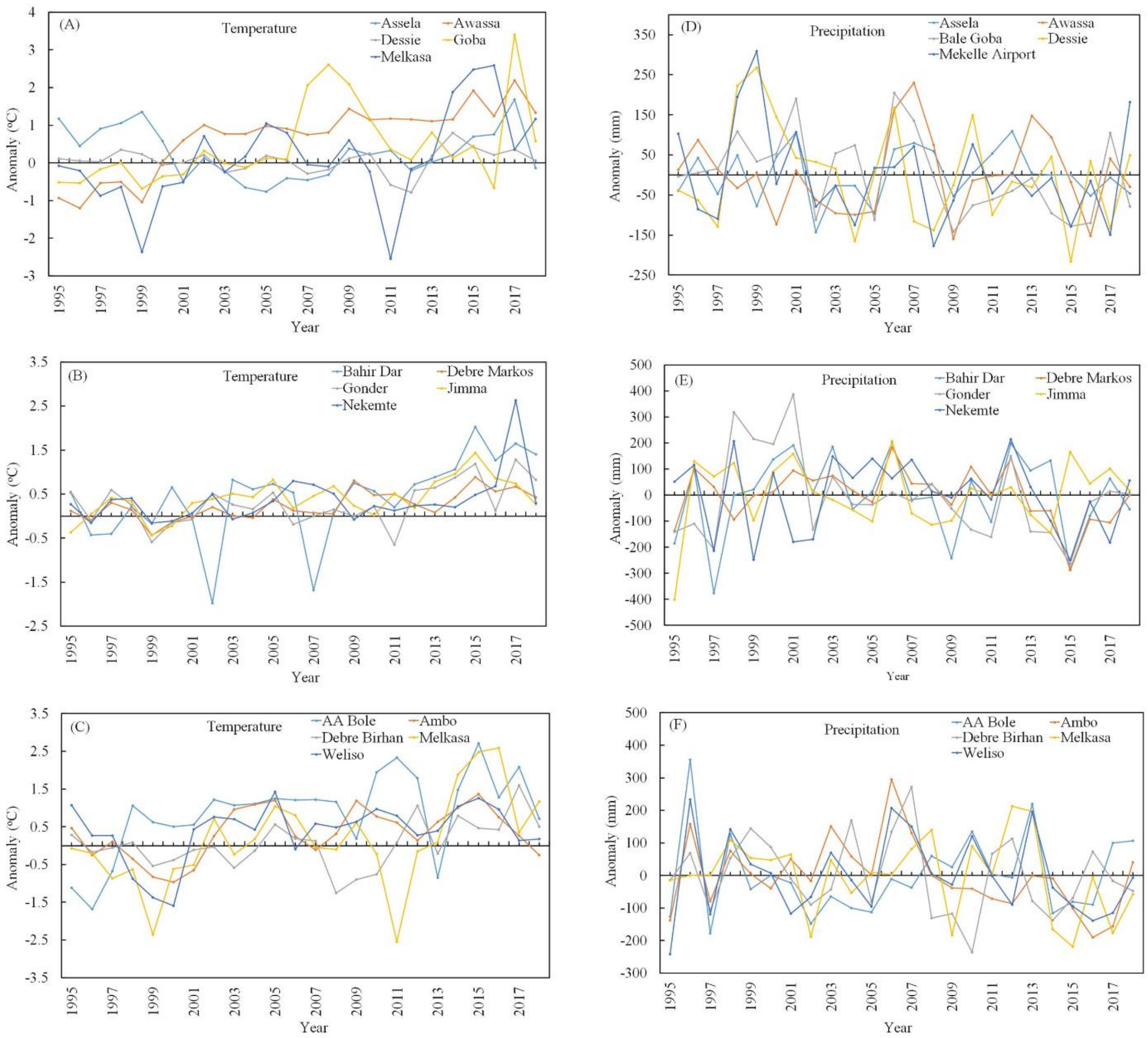

Fig. 4. Time series (1995–2018) of growing season mean temperature and total precipitation for each five representative metrological stations in the west (Debre Markos, Bahir Dar, Gonder, Nekemet, Jimma), east (Mekelle airport, Dese, Arsi, Goba and Awassa), middle (Addis Ababa Bole, Deber Birhan, Ambo, Melkassa, Weliso) parts of the country. The changes are estimated based on the mean value of the period 1995–2018.

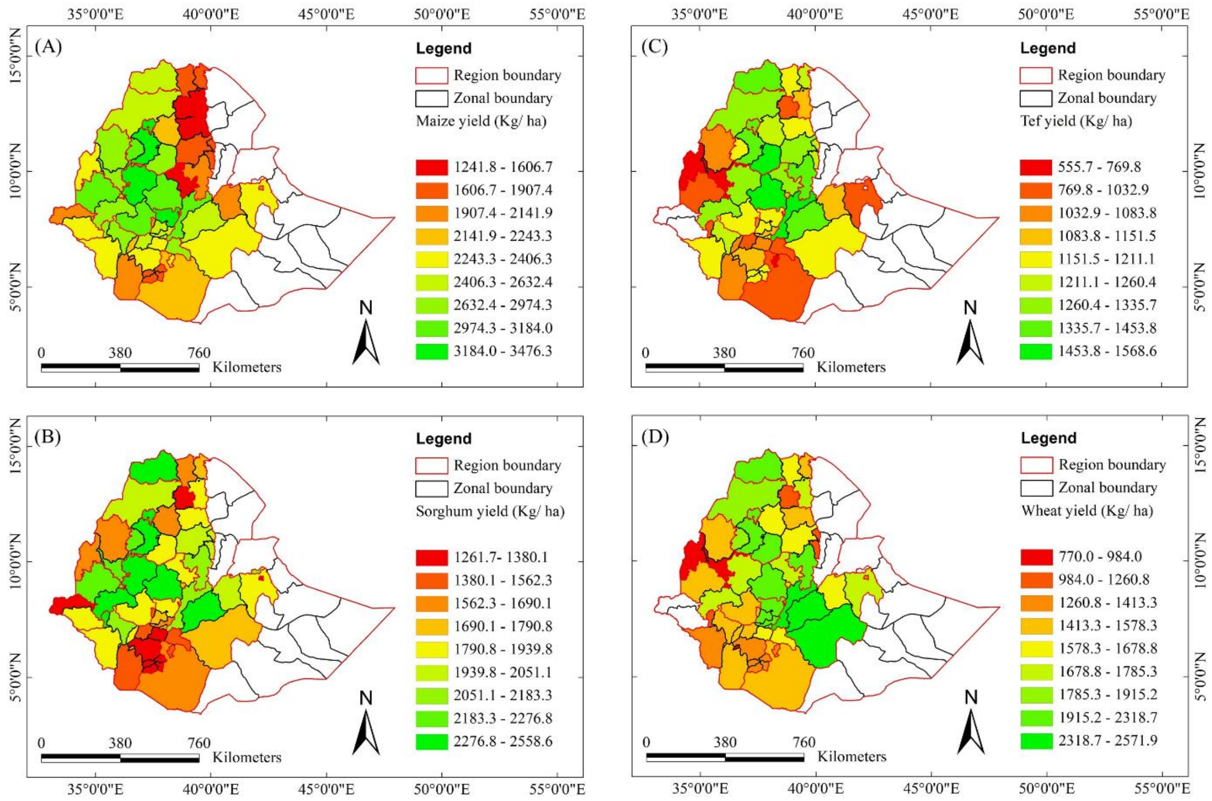

Fig. 5. Area coverage and average yields (1995–2018) of maize (A), sorghum (B), tef (C) and wheat (D) in the administrative zones of the major crop growing areas of Ethiopia.

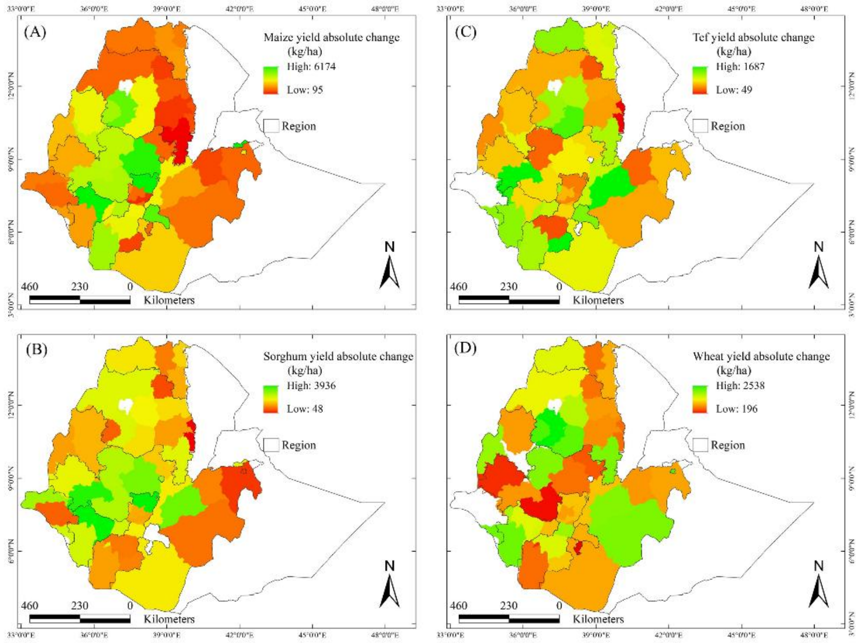

Fig. 6. Absolute changes in crop yields (kg/ha) for maize (A), sorghum (B), tef (C), and wheat (D) in the major crop growing areas of Ethiopia over the period 1995–2018.

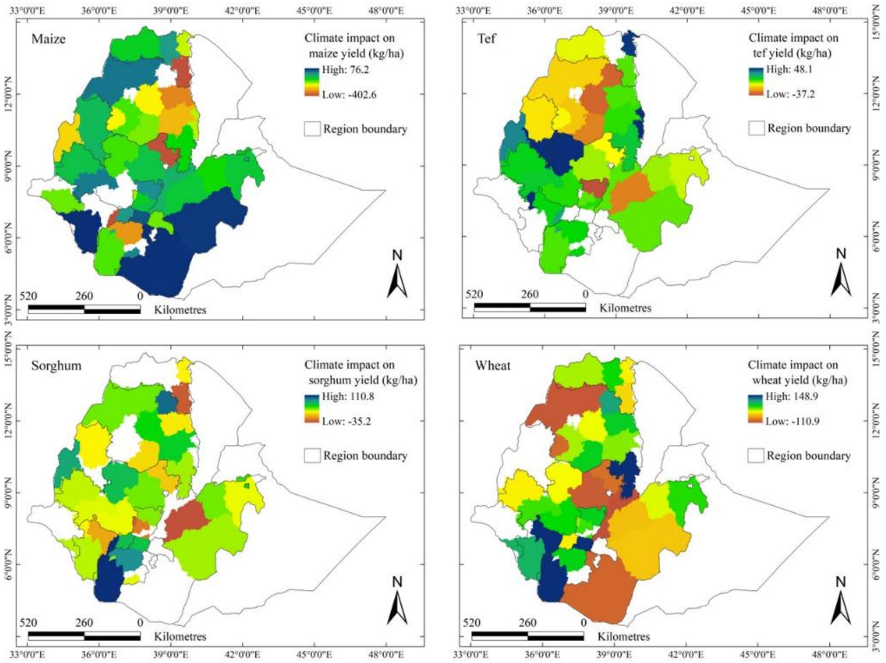

Fig. 7. Climate-induced crop yield impacts for maize, sorghum, tef, and wheat yields in the major crop growing areas of Ethiopia over the period 1995–2018. Values are represented as average yields (kg/ha) over the period 1995–2018. A positive sign indicates that the observed variability and trends in climate whether increasing or decreasing, have positively impacted crop yields, while a negative sign indicates that observed variability and trends in climate have negatively impacted crop yields.

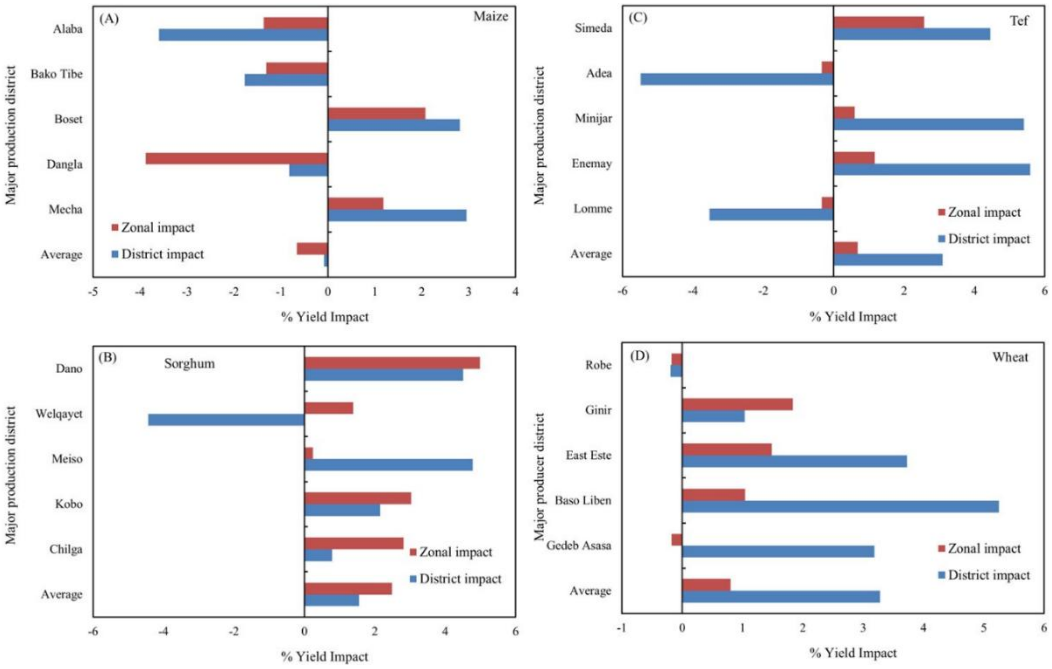

Fig. 8. Estimated net effects of the climatic trends on the trends in maize, sorghum, tef, and wheat yields over 1995–2018 across primary production districts and their corresponding administrative zones. Data are presented as a percentage of the average yield. The average values represent the weighted average estimates of the impacts of the climatic trends for each district and administrative zone.

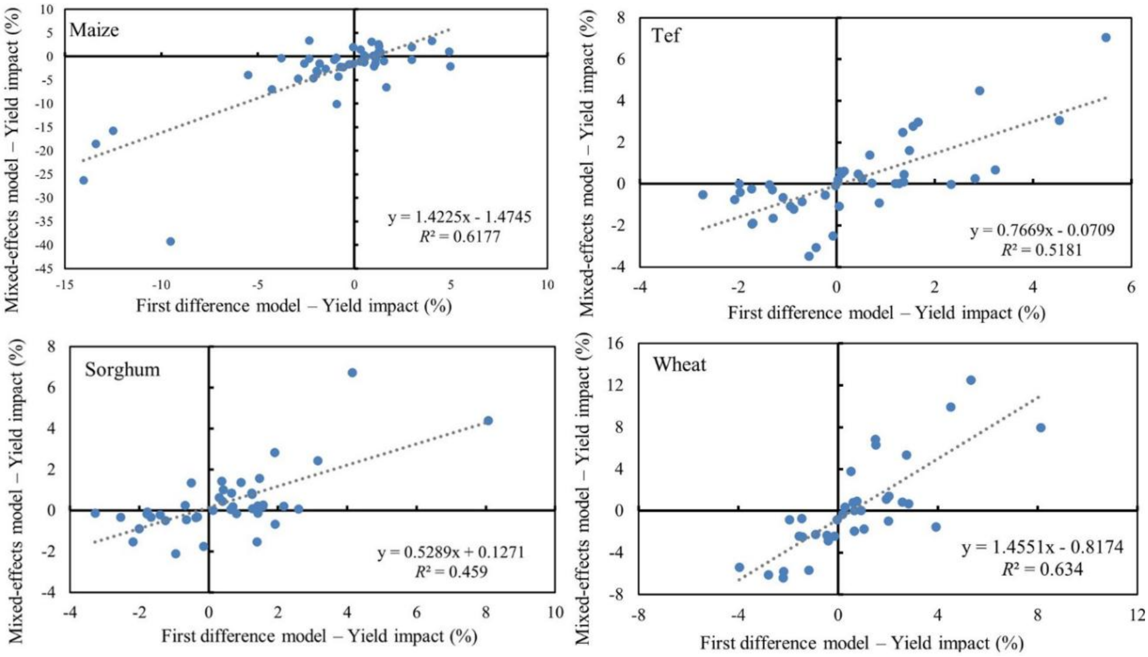

Fig. 9. Relationship between yield impacts estimated by mixed-effects model and yield impacts estimated by first difference regression model.

II: Supplementary Tables

Table 1. Meteorological data and trends for growing season (June to September) average air temperature and total precipitation time series over the period 1995–2018. Positive and negative values indicate increasing or decreasing trends, respectively. Bold numbers indicate significant trends (*p* <0.05).

Station	Lon	Lat	Elevation (m a.s.l.)	Temperature (ºC)			Precipitation (mm)		
				Mean	SD	Trend (ºC/year)	Mean	SD	Trend (mm/year)
A.A. BOLE	38.77	9.03	2443	17.3	1.08	**0.09**	782.09	119.83	1.47
A.A. OBSERVATORY	38.75	9.03	2441	16.95	1.21	**0.1**	806.73	125.61	4.45
ABIY ADI	39.03	13.67	2113	19.15	1.03	**0.105**	674.18	247.77	-5.49
ADABA	39.4	7.02	2396	15.56	0.46	-0.017	553.76	157.55	**7.51**
Adadi Mariam	38.5	8.63	2261	17.47	0.62	**0.043**	612.26	120.1	-0.57
Addis_Zemen_ Police_Stn	37.78	12.13	1954	19.46	1.24	0.004	1103.6	382.17	11.17
Adet	37.47	11.27	2235	17.66	0.99	**0.06**	877.15	115.36	1.91
Adi Gudom	39.52	13.25	2094	20.12	0.64	**0.042**	417.93	158.04	-1.44
Adigrat	39.46	14.27	2453	16.76	0.35	-0.002	340.93	87.48	-1.25
Adwa	38.8	14.17	2097	21.53	1.62	0.071	590.68	138.03	5.29
Agaro	36.59	7.85	1659	19.63	1.36	0.009	929.81	133.76	6.38
Aje	38.35	7.3	1840	20.37	1.02	0.056	277.61	99.39	-2.72
Akaki	38.82	8.9	2191	19.12	0.93	-0.021	672.93	103.72	-2.44
Alaba_Kulito	38.1	7.33	1795	20.25	1.3	**0.055**	414.34	74.38	-0.82
Alamata _Agr	39.68	12.52	1449	24.03	0.9	-0.016	399.06	111.19	-1.78
Alem Ketema	39.03	10.03	2352	14.41	1.08	**0.039**	791.29	102.67	2.11
Alem Teferi	35.22	8.91	1524	20.02	0.94	0.04	1043.4	221.19	**-16.6**
Alemtena	39.08	7.92	2238	17.47	0.46	0.001	448	68.34	1.26
Alibo	37.07	9.9	2342	16.5	0.72	-0.017	931.26	138.29	-3.46
Amba Giyorgis	37.59	12.76	2941	14.24	1.32	**0.139**	850.75	221.5	2.69
Ambo	37.87	8.97	2176	17.68	0.66	**0.036**	655.08	110.14	**-6.56**
Amed_Ber	37.08	11.88	1791	20.61	0.35	-0.008	1061	128.82	6.79
Andit Tid	39.71	9.81	3037	13.45	0.54	**0.041**	1080.5	164.03	-7.66
Anjeni	37.53	10.69	2453	14.13	0.44	-0.004	1534.7	202.8	-2.73
Arata	39.07	7.95	1812	18.78	0.45	0.007	463.2	71.7	0.84
Arba Minch	37.63	6.08	1175	23.09	1.5	-0.032	253.59	108.59	3.46
Arjo	36.5	8.76	2473	15.47	0.84	0.004	1003.3	170.41	-3.56
Arsi_Negele	38.65	7.35	1864	18.84	1.02	**0.071**	377.18	166.65	**-15.78**
Asebe_Teferi	40.87	9.07	1817	21.79	0.52	**0.027**	435.39	152.9	-3.8
Asela	39.13	7.95	2409	17.06	0.66	-0.009	532.12	64.42	-0.21
Asgori	38.2	8.97	2146	20.99	0.98	**0.092**	656.5	94.84	**-6.33**
Asosa	34.54	10.06	1573	19.82	0.72	-0.024	748.78	102.14	-4.47
Assendabo	37.23	7.77	1753	18.72	2.23	**-0.036**	724.32	98.43	2.38
Atnago	36.93	8.32	1713	18.45	1.39	0.014	1082.8	237.9	10.78
Awassa	38.48	7.05	1702	19.66	0.91	**0.101**	453.06	98.55	-0.81
Axum	38.72	14.11	2119	19.91	1.16	0.012	580.31	148.06	3.1
Azezo	37.42	12.55	2143	18.44	0.49	**0.033**	900.33	188.04	-3.82
Babile	42.35	9.21	1628	21.63	0.86	0.022	246.34	84	-1.81

Table 1. Continued, . . .

Station	Lon	Lat	Elevation (m a.s.l.)	Temperature (ºC)			Precipitation (mm)		
				Mean	SD	Trend (ºC/year)	Mean	SD	Trend (mm/year)
Baco	37.08	8.87	2011	17.74	0.48	**0.038**	686.38	109.09	1.95
Bahr Dar	37.38	11.6	1797	19.61	0.92	**0.073**	1158.1	155.08	-0.62
Bantu	38.37	8.61	2193	17.93	0.75	**0.042**	809.56	226.37	-3.22
Bedesa	40.77	8.9	1691	21.35	0.5	**0.043**	539.75	128.73	-7.25
Bichena	38.2	10.5	2520	16.17	1.17	0.013	824.32	146.95	**7.46**
Boditi_Sch	37.85	6.95	2060	17.79	0.47	0.014	545.16	206.78	-4.31
Bonga	36.23	7.22	1958	19.05	1.42	**0.067**	1129.6	181.92	-10.2
Bulki_Mindre	36.83	6.32	2265	21.31	2.25	**0.103**	498.61	106	-2.97
Butajira	38.37	8.12	2079	17.47	0.94	0.025	383.13	96.45	-2.44
C_Donsa	39.13	8.97	2317	17.38	0.63	**0.059**	662.93	109.19	5.06
Chagni	36.51	10.95	1642	17.65	1.36	**0.09**	1390.1	142.61	-4.05
Chencha	37.57	6.25	2729	16.35	1.27	**-0.112**	421.02	133.71	-4.34
Dabat	37.76	12.97	2589	14.14	0.91	**0.088**	697.33	99.27	-0.04
Dadim	38.22	4.93	1524	20.35	0.55	**-0.02**	99.72	37.97	0.03
Dagaga	38.97	7.6	2609	14.89	0.36	**0.021**	354.81	54.27	-1.97
Dangla	36.83	11.25	2130	17.73	1.12	**0.058**	1219.5	119.48	-2.31
Debark	37.89	13.15	2802	13.46	0.56	**0.041**	1236.7	218.39	6.08
Debre Berhan	39.58	9.63	2855	12.77	0.63	**0.032**	753.71	119.21	-3.51
Debre Markos	37.73	10.34	2462	15.6	0.32	**0.027**	947.33	101.76	-4.86
Debre Tabor	38.01	11.85	2689	14.8	0.36	**0.026**	1089.9	165.7	4.06
Debre Werk	38.16	10.66	2532	16.58	0.94	0.026	651.72	98.41	-1.44
Debre Zeit	36	10.17	910	19.64	0.57	-0.011	1564.6	468.08	8.95
Dedo	36.88	7.51	2262	16.9	1.05	**0.082**	901.53	298.15	**-30.6**
Degollo/Dongoro	35.83	9.2	1841	18.9	0.35	0.011	1231.6	208.84	3.18
Dejen	38.15	10.17	2420	17.19	1.24	0.025	945.12	207.45	**-17.14**
Delo Sebro	40.47	7.25	2216	17.68	2.06	**0.275**	298.03	101.77	-4.01
Dembecha	37.47	10.57	2128	17.25	0.83	-0.033	1212.9	254.03	8.02
Dembi	36.45	8.07	1753	20.58	1.08	**0.067**	1029.5	147.59	5.03
Dembidolo	34.8	8.53	1845	18.85	0.54	**0.051**	1183.9	498.34	**-42.87**
Derba	38.63	9.43	1760	19.72	0.94	0.024	724.85	84.79	-0.9
Dese	39.64	11.12	2509	17.19	0.33	0.004	785.23	125.04	-4.87
Dilela	38.04	8.63	2388	16.73	0.83	0.036	777.13	109.58	0.27
Dinsho	39.77	7.1	3070	11.66	0.63	**0.045**	818.1	403.41	-4.36
Dire Dawa	41.86	9.6	1196	23.45	1.19	**0.05**	292	76.09	-0.74
Dizi	35.6	8.4	1655	19.45	0.93	**0.03**	989.01	150.86	-5.67
Dodola	39.18	6.98	2485	15.76	0.44	-0.019	536.03	115.19	0.84

Table 1. Continued, . . .

Station	Lon	Lat	Elevation (m a.s.l.)	Temperature (ºC)			Precipitation (mm)		
				Mean	SD	Trend (ºC/year)	Mean	SD	Trend (mm/year)
Durame	37.89	7.24	2032	19.69	0.93	0.044	525.39	99.73	**3.29**
Efeson	39.95	10.34	1478	22.55	1.26	**0.103**	703.31	187.48	-2.02
Enfranz	37.68	12.18	1930	20.3	0.94	**0.063**	904.27	163.83	13.05
F. Selam	37.16	10.42	1561	19.87	0.58	**0.031**	807.59	131.67	-1.65
Fincha	37.37	9.22	2389	16.8	0.91	**0.082**	936.81	127.47	**8.23**
Fiseha_Genet	38.2	6.12	1966	17.09	0.82	**0.045**	402.28	126.53	2
Gasera	40.19	7.37	2355	15.46	0.68	**0.059**	432.89	89.55	-2.66
Gato	37.41	5.56	1356	24.58	0.86	-0.026	184.77	74.82	2.03
Gebere_Guracha	38.42	9.82	2538	15.46	0.9	**-0.052**	1005.1	117.46	5.66
Gedo	37.43	9.05	2747	15.88	1.28	**0.106**	620.09	183.19	**15.44**
Gelemso	40.51	8.81	1762	21.43	0.52	**0.032**	548.23	118.99	-2
Gerese	37.29	5.93	2358	17.6	1	0.025	538.75	272.23	1.38
Gidole	37.35	5.65	2468	17.92	1.94	**-0.206**	290.03	128.61	**8.81**
Ginchi	38.12	9.03	2261	18.27	0.7	**0.064**	724.96	179.35	-5.48
Ginir	40.71	7.15	1953	18.32	1.58	0.058	179.47	65.32	1.57
Girawa	41.85	9.13	2342	15.74	0.67	0.008	443.74	111.87	-3.44
Goba Robe	39.97	7.02	2697	15.52	1.07	**0.052**	386.77	101.02	-5.65
Gobesa	39.49	7.62	2407	17.31	0.93	0.009	515.72	138.27	-5.12
Gohatsion	38.23	10	1928	17.7	1.05	**-0.08**	930.32	148.41	5.61
Gonder A.P	37.42	12.55	2120	18.44	0.49	**0.033**	900.33	188.04	-3.82
Gore	35.53	8.15	2034	17.7	0.31	**0.02**	1075.5	162.14	-2.34
Gorgora	37.29	12.24	1826	20.06	0.86	-0.039	843.1	136.19	1.45
Guder	39.78	8.95	1056	18.68	0.79	-0.012	625.42	126.89	**-7.07**
Guliso	35.52	9.2	1511	18.78	1.26	-0.034	996.45	151.58	-6.38
Gununo	37.64	6.93	2009	18.46	0.52	-0.015	533.12	168.18	-0.72
Gursum	42.38	9.35	1822	18.68	0.64	0.025	314.09	72.14	-2.79
Hagere Selam	39.15	13.65	2689	16.09	0.52	**0.035**	529.44	98.68	-0.85
Harer	42.11	9.31	2017	19.2	0.55	0.018	311.8	93	-3.71
Hartisheik	43.9	9	1385	23.47	0.55	-0.003	163.55	103.39	-0.17
Hawzien	39.45	13.9	2123	19.33	0.55	**0.034**	388.83	91.37	1.22
Hayk	39.68	11.32	2026	20.78	0.31	**-0.017**	773.44	151.52	-0.93
Hibino	39.01	10.46	1920	18.37	0.95	**0.093**	634.13	107.97	-5.77
Hirna	41.12	9.22	1941	20.14	0.99	0.006	536.1	107.96	**-6.98**
Holeta	38.3	9	2155	20.15	0.99	**0.078**	641.2	80.64	**-4.83**
Humera	36.6	14.27	596	26	1.34	-0.004	422.92	118.36	-1.19

Table 1. Continued, . . .

Station	Lon	Lat	Elevation (m a.s.l.)	Temperature (ºC)			Precipitation (mm)		
				Mean	SD	Trend (ºC/year)	Mean	SD	Trend (mm/year)
Hunde Lafto	41	9.11	2006	19.54	0.45	0.023	437.65	90.36	-0.87
Huruta	39.37	8.15	1958	19.11	0.37	0.022	517.92	107.46	-4.31
Ibnat	38.05	12.13	2177	19.8	1.2	0.102	873.03	198.47	6.24
Idaga Hamus	39.56	14.18	2652	16.29	0.59	0.001	344.82	115.05	-4.01
Indibir	37.93	8.12	2099	18.2	0.7	-0.038	800.23	182.2	**13.38**
Jijiga	42.78	9.33	1633	20.27	1.15	0.018	315.27	74.16	**-6.68**
Jimma	36.84	7.67	1712	19.59	0.43	0.034	954.79	130.85	-1
Key_Afer	36.73	5.52	1585	22.37	1.41	-0.07	281.35	97.32	-1.96
Kimoye	38.35	9.03	2244	16.9	0.73	0.08	724.96	103.7	-1.66
Koka_Dam	39.17	8.42	1664	22.3	0.9	0.009	468.63	147.32	5.9
Kombolcha	39.74	11.08	1823	21.45	0.4	0.03	675.58	94.08	1.11
Korem	39.52	12.49	2469	17.23	0.46	0.001	585.72	144.36	-2.02
Kulmesk	39.21	11.94	2347	15.86	0.51	0.019	491.88	121.41	**-7.06**
Kulubi	41.67	9.43	2222	19.95	1.42	**0.016**	490.02	130.39	-3.01
Lalibela	39.02	12.04	2254	16.89	0.76	**0.058**	685.28	153.98	-4.28
Lemi	38.9	9.82	2661	15.36	0.73	0.015	931.89	198.97	-7.48
Maybar	39.66	11	2519	19	0.52	0.02	696.67	129.71	6.01
Maychew	39.55	12.79	2406	20.17	0.64	**0.032**	421.13	122.03	-2.85
Mega	38.25	4.05	1602	21.54	0.77	**0.042**	41.34	64.72	0.93
Mehoni	39.65	12.79	1748	23.87	0.72	**0.035**	351.36	102.89	-3.01
Mekaneyes us	38.07	11.65	2429	16.47	0.37	0.001	1002.9	151.1	-2.34
Mekele Air port	39.47	13.49	2079	19.24	0.4	**0.02**	429.15	118.2	-2.98
Meksenyit	37.57	12.35	1905	20	0.39	0.001	868.44	158.5	**-7.83**
Melkasa	39.34	8.4	1520	21.79	1.22	**0.081**	492.15	118.58	-2.13
Mendi	35.1	9.8	1680	20.35	0.74	-0.009	1444.1	355.81	-19.37
Mendida	38.32	9.65	1811	18.34	0.76	**-0.054**	842.75	92.98	1.37
Metu	35.59	8.3	1683	18.57	0.8	**-0.059**	1144.9	193.31	-9.13
Minjar	39.43	8.93	1795	20.77	0.99	**0.09**	555.68	121.2	1.1
Mojo	39.12	8.62	1768	20.02	0.83	0.023	686.31	148.72	6.58
Motta	37.87	11.08	2412	17.46	1.2	-0.012	829.31	109.56	-0.26

Table 1. Continued, . . .

Station	Lon	Lat	Elevation (m a.s.l.)	Temperature (ºC)			Precipitation (mm)		
				Mean	SD	Trend (ºC/year)	Mean	SD	Trend (mm/year)
Nefas Mewucha	38.45	11.73	2949	13.6	0.6	**0.05**	977.26	231.77	-2.57
Nekemte	36.55	9.09	2109	17.13	0.56	**0.021**	1156	142.13	-3.78
Neshi	37.2	9.65	2338	17.35	0.44	**0.03**	1165.4	201.29	1.29
Nopa	35.6	8.28	1769	18.57	0.8	**-0.06**	1144.9	193.31	-9.13
Ogolcho	39.03	8.07	1735	20.73	0.8	**0.014**	383.03	100.11	-0.45
Robe	39.63	7.86	2423	15.83	0.5	-0.011	422.39	67.78	-1.92
Samre	39.2	13.17	1850	22.03	0.91	**0.103**	438.81	116	-2.11
Sekota	39.03	12.63	2252	19	0.58	**0.026**	465.59	107.07	1.42
Shambu	37.05	9.57	2768	15.66	0.4	0.008	1501.4	191.17	-2.8
Shebe	36.52	7.52	1791	18.92	0.88	**0.057**	833.23	197.33	3.99
Shebel_Berenta_Yeduha	38.33	10.33	2129	17.91	0.69	0.031	749.8	99.04	**6.72**
Shire	37.78	14.33	1042	25.51	0.87	0.005	568.44	137.52	-1
Shishinda	35.87	7.22	1971	16.51	1.09	**-0.09**	685.78	139.54	-6.66
Sibusire	36.85	9.03	1797	19.64	0.45	**0.03**	915.22	126.24	3.03
Simada	38.14	11.29	2225	17.32	1.18	0.004	805.27	103.88	-3.36
Sirinka	39.62	11.55	2002	18.9	0.7	0.036	742.55	175.16	-2.58
Soddo	37.76	6.86	2022	18.21	0.33	0.003	516.08	185.97	-1.5
Sululta	38.73	9.18	2566	13.29	1.51	**0.159**	1012.8	189.8	2.97
Supe	35.63	8.47	1390	20.83	0.68	0.015	1119.3	222.63	8.39
Tikur_Enchine	37.63	8.78	2485	16.08	1.19	**0.119**	886.65	169.21	6.93
Tulu Bolo	38.21	8.66	2184	17.27	0.89	**0.051**	796	179.56	-7.76
Uka	35.18	8.23	1609	19.77	0.67	-0.009	939.23	150.53	-6.98
Wegel Tena	39.22	11.59	2962	15.32	1.07	0.019	662.54	156.84	-6.85
Weliso	37.97	8.54	2040	18.36	0.75	0.026	953.57	123.53	-2.25
Wendo Genet	38.61	7.04	1738	19.25	0.61	**0.043**	441.22	131.97	0.03
Wereta_Add	37.68	11.92	1802	19.64	1.04	**0.059**	1115.2	217	-1.2
Worabe	38.1	7.8	2054	17.72	0.84	0.011	609.95	127.32	-0.98
Wukro	39.6	13.78	1988	20.24	0.89	0.024	465.76	132.24	-4.55
Wulbareg	38.13	7.75	2004	18.03	0.84	0.001	622.72	143.49	-3.12
Yergalem	38.38	6.75	1692	18.85	0.72	**0.026**	426.94	118.74	-0.66
Yetemen	38.13	10.33	2408	17.29	1.06	**0.098**	833.91	249.1	-4.33
Yirba Moda	38.71	6.21	2571	15.6	0.35	0.011	548.83	191.32	**11.09**
Yirga Chefe	38.2	6.16	1847	17.79	1.23	**0.035**	418.08	153.48	0.52
Yubdo	35.12	9.03	1865	18.58	0.56	0.007	958.25	220.17	**-22.29**
Zege	37.31	11.69	1799	19.85	0.5	**0.048**	1428.2	234.07	6.29

Table 2. Trends and variation of yield for maize, sorghum, tef and wheat in productive districts of Ethiopia for the period 1995–2018. Bold numbers indicate significant trends (*p* <0.05).

		Maize (kg/ha)			Sorghum (kg/ha)				
Zone	District	Mean	SD	Trend (kg/ha/year)	Zone	District	Mean	SD	Trend (kg/ha/year)
Awi	Ankasha Guagusa	3515	921	140	North Gonder	Adiarikay	2231	614	57
Awi	Dengila	3679	836	114	North Gonder	Chilga	2444	1043	95
West Gojjam	Bahir dar zuriya	2712	812	-20	North Gonder	Debark	2118	668	93
West Gojjam	Bure	3513	792	79	North Gonder	Dembia	1951	569	77
West Gojjam	Dembecha	3908	1495	247	North Gonder	Gondar Zuriya	1757	634	81
West Gojjam	Mecha	3904	1212	124	North Gonder	Metema	2644	644	92
West Gojjam	North Achefer	3818	1082	161	North Gonder	Quara	2507	650	81
West Gojjam	South Achefer	3646	959	154	North Shewa	Merhabete	2011	697	12
West Gojjam	Wenberma	4320	1415	151	North Shewa	Mida Oromo	2353	530	84
East Shewa	Adami Tulu Jido	2992	963	076	North Wollo	Habru	1573	348	-14
East Shewa	Boset	2479	1129	049	North Wollo	Kobo	1996	423	0
East Shewa	Dugda	3687	992	100	Oromia	Bati	2221	467	46
East Wellega	Gida Keremu	2876	797	-28	South Wollo	Kalu	1450	579	24
East Wellega	Limu	4246	1410	12	East Hararge	Girawa	1789	453	20
East Wellega	Sibu Sire	4203	1466	148	West Hararge	Meiso	3884	1795	196
Guji	Bore	2908	1184	76	West Hararge	Mesela	1971	783	8
Horo Gudru Wellega	Abe Dengoro	3280	953	-38	West Shewa	Abuna Gindeberet	2515	539	47
Horo Gudru Wellega	Gudru	3915	949	80	West Shewa	Dano	3087	889	109
Ilu Aba Bora	Darimu	3986	1631	204	North Western	Asegede Tsimbila	2196	671	49
Jimma	Limu Kosa	3442	949	22	North Western	Tahitay Adiyabo	2679	726	108
Qeleme Wellega	Hawa Gelan	2334	857	-101	North Western	Tselemt	2568	827	113
West Arsi	Arsi Negele	3531	955	-62	South Tigray	Rya Azebo	2176	667	10
West Shewa	Bako Tibe	2807	937	102	Western Tigray	Qafta Humera	2315	644	-13
West Shewa	Dano	3585	1151	162	Western Tigray	Tsegede	2892	813	-9
Alaba	Alaba	3399	1364	160	Western Tigray	Welqayet	3338	719	58

Table 2. Continued, . . .

Zone	District	Tef (kg/ha)			Wheat (kg/ha)				
		SD	Mean	Trend (kg/ha/year)	Zone	District	SD	Mean	Trend (kg/ha/year)
Awi	Ankasha Guagusa	1670	363	-8	East Gojjam	Baso Liben	2606	1094	198
East Gojjam	Aneded	1230	349	-13	North Gonder	Jan Amora	2166	438	41
East Gojjam	Awabel	1653	318	14	North Gonder	Wegera	1332	555	-19
East Gojjam	Dejen	1681	618	60	North Shewa	Minjar Shenkora	2296	656	61
East Gojjam	Enarj Enawuga	1802	531	32	South Gonder	Misrak Este	1670	462	71
East Gojjam	Enemay	1875	340	35	Arsi	Digeluna Tijo	2418	808	34
East Gojjam	Huletej Enese	1839	560	82	Arsi	Hitosa	3501	827	61
East Gojjam	Shebel Berenta	1731	535	39	Arsi	Limuna Bilbilo	2885	891	106
North Gonder	Alefa	1811	491	1	Arsi	Munesa	2712	1025	35
North Shewa	Minjar Shenkora	1603	498	62	Arsi	Robe	3197	1320	215
South Gonder	Dera Gonder	1304	345	27	Arsi	Shirka	3302	812	74
South Gonder	Misrak Este	1652	368	61	Arsi	Sire	2822	936	131
South Gonder	Simada	1493	508	92	Arsi	Tiyo	2144	732	38
South Wollo	Wogidi	1603	317	6	Bale	Agarfa	2913	825	115
West Gojjam	Yilmana Densa	1438	385	26	Bale	Gasera	2229	655	86
East Shewa	Adama	1116	326	-5	Bale	Ginir	1767	696	90
East Shewa	Adea	1307	429	25	Bale	Sinana	2070	919	-12
East Shewa	Lomme	2355	846	60	East Shewa	Dugda	2149	561	51
North Shewa	Dera Shewa	1372	489	39	East Shewa	Gimbichu	2488	1091	22
North Shewa	Were Jarso	1451	421	12	South West Shewa	Shewa Ameya	2480	496	27
South West Shewa	Becho	1069	410	2	West Arsi	Adaba	2430	635	37
South West Shewa	Sebeta Hawa	1770	391	29	West Arsi	Arsi Negele	2681	551	10
South West Shewa	Ameya	1730	382	58	West Arsi	Dodola	2789	667	-12
West Shewa	Abuna Gindeberet	1716	340	33	West Arsi	Gedeb Asasa	3265	813	98
West Shewa	Adea Berga	1761	476	19	South Tigray	Enderta	1708	542	-2

Table 3. Meteorological data and trends for growing season (June–September) average air temperature and total precipitation time series over the period 1995–2018 in different administrative zones of Ethiopia. Bold numbers indicate significant trends (p <0.05). Positive or negative values indicate an increasing or a decreasing trend, respectively.

No.	Administrative region	Administrative zone	Temprature (ºC)			Precipitation (mm)		
			Mean	STD	Trend (ºC/year)	Mean	STD	Trend (mm/year)
2	Tigray	Western Tigray	20.72	0.53	0.03	658.68	121.38	1.71
3	Tigray	Central Tigray	19.23	0.65	**0.05**	525.75	140.06	2.02
4	Tigray	East Tigray	18.44	0.37	0.01	397.09	109.01	-1.24
5	Tigray	South Tigray	20.36	0.57	**0.02**	429.85	121.99	-0.7
7	Amhara	North Gondar	18.13	0.46	**0.04**	934.34	113.11	1.35
8	Amhara	South Gondar	17.28	0.35	**0.03**	946.56	124.27	2.76
9	Amhara	North Wollo	17.34	0.38	**0.02**	645.56	129.28	-5.34
10	Amhara	South Wollo	18	0.29	**0.02**	712.89	111.44	0.67
11	Amhara	North Shewa (Amhara region)	16.65	0.42	**0.05**	771.37	101.14	-0.79
12	Amhara	East Gojam	16.8	0.42	0.01	902.35	92.26	-1.03
13-15	Amhara	West Gojjam	18.05	0.31	**0.02**	1106.56	107.11	0.79
16	Amhara	Waghemra	18.75	0.4	**0.04**	551.05	121.28	-0.79
17	Amhara	Awi	17.81	0.7	**0.05**	1241.23	111.89	-2.43
18	Amhara	Oromia Special Zone	19.98	0.51	**0.04**	701.37	119.93	0.38
19	Amhara	Bar Dar Sp. Zone	19.56	0.87	**0.07**	1151.07	158.98	0.00
20	Oromia	West Welega	19.24	0.28	0.00	1109.55	163.99	**-14.22**
21	Oromia	East Welega	17.28	0.26	**0.02**	1079.75	101.36	0.09
22	Oromia	Ilu Ababora	18.92	0.27	0.00	1048.54	94.36	-3.15
23	Oromia	Jimma	18.66	0.53	**0.03**	905.72	72.98	0.86
24	Oromia	West Shewa	17.59	0.53	**0.05**	774.09	85	0.62
25	Oromia	North Shewa (Oromia region)	16.74	0.38	**0.02**	826.89	100.69	0.59
26	Oromia	East Shewa	19.23	0.38	**0.03**	522.44	68.14	-0.52
27	Oromia	Arsi	17.74	0.26	**0.02**	475.03	66.77	-1.4
28	Oromia	West Harerge	20.19	0.36	**0.03**	495.74	75.97	-3.81
29	Oromia	East Harerge	19.59	0.43	**0.02**	334.72	68.71	-3.5
30	Oromia	Bale	16.65	0.63	**0.06**	430.69	75.18	0.31
31	Oromia	Borena	19.2	0.26	-0.01	303.77	66.96	2.67
32	Benishangul Gumuz	Metekel	18.84	0.48	0.02	1257.19	133.35	0.72

Table 3. Continued, . . .

No.	Administrative region	Administrative zone	Temprature (ºC)			Precipitation (mm)		
			Mean	STD	Trend (ºC/year)	Mean	STD	Trend (mm/year)
33	Benishangul Gumuz	Asosa	19.61	0.43	-0.01	1073.82	135.2	-8.95
34-37	Benishangul Gumuz	Kemashi	18.78	0.34	0.01	1233.42	128.04	-2.74
38	SNNP	Gurage	17.94	0.36	**0.02**	638.91	93.6	1.74
39-40	SNNP	Hadiya	18.61	0.48	0.01	582.55	85.25	0.68
41	SNNP	Kembata - Tembaro	19.28	0.58	**0.04**	495.85	78.32	0.57
42	SNNP	Sidama	18.23	0.45	**0.03**	427.42	125.97	1.32
43	SNNP	Gedio	17.61	0.82	**0.03**	427.42	125.97	1.32
44	SNNP	Wolayita	18.45	0.27	0.01	517.03	144.98	-1.25
45	SNNP	South Omo	20.69	0.58	**-0.04**	386.1	87.29	-0.5
46	SNNP	Sheka	18.48	0.41	-0.02	981.98	98.27	-4.95
47	SNNP	Keffa	18.41	0.61	0.01	981.98	98.27	-4.95
48	SNNP	Gamo Gofa	19.47	0.51	0.01	456.44	112.26	-0.28
49	SNNP	Bench - Maji	18.93	0.45	-0.01	767.21	82.67	-4.64
50	SNNP	Yem Special	19.71	0.66	**-0.04**	734.5	81.92	3.16
51	SNNP	Amaro Special Woreda	19.71	0.66	**-0.04**	356.21	99.46	2.25
52	SNNP	Burji Special Woreda	20.32	0.63	**-0.05**	313.09	90.93	2.12
53	SNNP	Konso Special Woreda	18.92	0.58	**0.03**	280.97	91.64	2.41
54	SNNP	Derashe Special Woreda	17.92	1.93	**-0.2**	303.1	103.19	4.2
55	SNNP	Dawro	18.89	0.94	**0.08**	666.79	103.55	-2.93
56	SNNP	Basketo Special	21.84	1.18	0.03	511.09	93.25	-1.97
57	SNNP	Konta Special	18.24	0.84	**0.04**	781.06	92.06	-4.8
58	Gambella	Nuware	19.07	0.25	0.01	1052.97	174.41	**-17.42**
59	Gambella	Agnuwak	18.76	0.32	-0.01	1010.26	113.81	**-8.94**
60	Harari	Harari	19.52	0.41	**0.03**	309.78	74.73	-2.04
62	Dire Dawa	Dire Dawa	21.47	0.7	**0.04**	332.91	72.96	-0.38

Table 4. Effects of fixed and random variables on maize, sorghum, tef and wheat yields at selected district level. T: Temperature (°C), P: Precipitation (mm), R: Solar radiation (w/m^2); T^2: Squared terms for Temperature, and P^2: Square terms for precipitation.

Maize						
Fixed effects					Random effects	
Variable	Coef.	Std.Error	t-value	p-value	Variance	StdDev
(Intercept)	7.4	0.033	226.656	0.000	0.066	0.256
Year	0.046	0.003	14.18	0.000	0.0003	0.017
T	-0.002	0.010	-0.719	0.472	0.0008	0.029
T^2	-0.012	0.006	-2.17	0.030	0.0007	0.026
P	-0.006	0.010	-1.636	0.102	0.0007	0.026
P^2	-0.015	0.007	-2.015	0.044	0.0002	0.005
R	-0.019	0.010	-1.987	0.047	0.0003	0.006
Residual					0.0350	0.188

Sorghum						
Fixed effects					Random effects	
Variable	Coef.	Std.Error	t-value	p-value	Variance	StdDev
(Intercept)	7.116	0.049	145.98	0.000	0.5353	0.287
Year	0.051	0.003	17.49	0.000	0.111	0.012
T	0.002	0.009	1.68	0.094	0.124	0.016
T^2	-0.008	0.006	-2.46	0.014	0.093	0.009
P	0.007	0.010	1.87	0.062	0.198	0.039
P^2	-0.015	0.008	-2.02	0.044	0.164	0.027
R	-0.001	0.009	-1.67	0.095	0.147	0.022
Residual					0.442	0.195

Table 4. Continued, . . .

Tef						
Fixed effects					Random effects	
Variable	Coef.	Std.Error	t-value	p-value	Variance	StdDev
(Intercept)	6.693	0.038	175.84	0.000	0.449	0.201
Year	0.046	0.003	15.15	0.000	0.109	0.012
T	-0.007	0.010	-1.68	0.093	0.179	0.032
T^2	-0.020	0.006	-3.31	0.001	0.105	0.011
P	0.007	0.010	1.66	0.097	0.178	0.032
P^2	0.005	0.008	2.11	0.035	0.183	0.034
S	0.008	0.010	1.67	0.095	0.210	0.044
Residual					0.453	0.205

Wheat						
Fixed effects					Random effects	
Variable	Coef.	Std.Error	t-value	p-value	Variance	StdDev
(Intercept)	7.134	0.048	149.05	0.000	0.506	0.256
Year	0.043	0.004	11.617	0.000	0.130	0.017
T	0.005	0.011	1.38	0.167	0.170	0.029
T^2	-0.024	0.007	-3.24	0.001	0.161	0.026
P	-0.007	0.009	-2.42	0.016	0.161	0.029
P^2	-0.005	0.007	-1.68	0.093	0.069	0.005
S	0.022	0.008	2.66	0.008	0.079	0.006
Residual					0.434	0.188

III. Supplementary methods

A. CSA sampling methodology

In Ethiopia, the primary administrative division is known as a region, also referred to as a regional state (Supplementary Fig. 1). According to data from the [1], there are eleven regional states and two chartered cities (Addis Ababa and Dire Dawa). Regions are further divided into administrative zones, constituting the second level of subdivision in Ethiopia, positioned below regions and above districts (woredas). Although the number of zones varies, most regions typically encompass around five to twelve zones, with the largest region, Oromia, having over 21 zones. Administrative zones, in turn, consist of districts (woredas), with each zone comprising approximately more than 15 districts. The district serves as the administrative unit where crucial agricultural development decisions are made. Consequently, district and zonal

levels agricultural production data are important for facilitating the planning, implementation, monitoring, and evaluation of agricultural interventions at that levels.

The Ethiopia's Central Statistics Agency (CSA) Agricultural Sample Survey (AgSS) data set is an extensive agricultural survey that typically evaluates over 500,000 agricultural plots, 38,000 households in 1,850 Enumeration Areas (EAs) that reside in approximately 540 agricultural districts. The CSA determines particular EA's as individual groups of approximately 150−200 households based on geographical considerations. Next, the CSA amalgamates EAs according to a methodology that reflects broader zonal, administrative areas. Production estimates are determined at the agricultural household level by projecting the sampled household production onto the overall projected agricultural population of the area. The CSA employs a stratified sampling technique that selects EAs according to a CSA algorithm that chooses a random sample of twenty households within each of their identified EAs. In addition, EAs are chosen for a three-year period of estimation for the AgSS survey.

The CSA samples both area and production for the AgSS. When sampling at the EA level, the CSA takes a sample crop cut from a maximum of five farmers, averages the yields across the number of crop cuts, and then projects this average for all of the farmer's area plots at the EA level. In other words, while the CSA takes area measurements for all sampled agricultural plots, they do not measure yield estimates from each plot. This means that for a sampled minimum of twenty households within a district, five crop cuts at most ultimately determine the productivity for an entire district for any given year. Since every plot is assumed to have the same yield in a given EA, there are very few yield samples per district.

B. Development of Administrative zonal (AZ)- and district-based crop yield and climate data

For the purpose of exploration and investigation of impacts of climate on the crop yields, it is crucial that both datasets (climate and crop yields) are at the same spatial and temporal scales, hence rendering them suitable for inter-comparison. These scales have to be appropriate in terms of space and time so that the resulting climatic impacts are represented in a detailed, consistent, and relatable manner. In this study, administrative zone and district have been chosen as an appropriate scale to investigate and report the findings. This is because information of crop yields and other management practices are mainly reported on these levels. Thus, quantifying climate impacts on crop production on AZ and district basis results in increased

potential for the application of these findings by the state and federal agencies working in the disciplines of agriculture and natural resource conservation.

To ensure the reliability of the climate dataset, we implemented rigorous quality control procedures. Temperature and precipitation data were obtained from 175 meteorological stations operated by the Ethiopian National Meteorological Agency (NMA). Gaps in station-level records were filled using gridded reconstructions also provided by the NMA. The complete station dataset was then interpolated using the Inverse Distance Weighting (IDW) method in ArcGIS 10.2 at a spatial resolution of 1 km. The interpolated data were subsequently aggregated to both administrative zone and district levels.

Validation of the interpolated data against observed station records for the period 2010–2018 revealed strong correlations, confirming the reliability of the gridded datasets for further analysis (see Supplementary Table 5). Solar radiation data were similarly disaggregated to a 1 km resolution using bilinear interpolation, and administrative zone- and district-level values were computed using the Zonal Statistics tool in ArcMap 10.2.

The resulting climate database includes zone- and district-level averages of growing season mean temperature, total precipitation, and monthly total solar radiation. These datasets cover up to 62 administrative zones, specifically 57, 62, 51, and 44 zones for maize, sorghum, teff, and wheat, respectively, and 25 selected high-potential districts for each crop, totaling 92 districts across all four crops. The climate data span the period 1995–2018, aligning with the available crop yield data.

Crop yield data at national, AZ and district levels were sourced from the CSA [1]. Given variations in data completeness and consistency at the district level, we restricted our analysis to districts with at least 21 years of data and less than 10% missing values. We focused on productive districts identified by Warner et al. [8] to ensure relevance and data robustness. To maintain spatial consistency, missing values in the AZ- and district-level datasets were imputed using corresponding national-level and AZ-level values, respectively.

C. Gridding and validation of climate data

C1. Inverse distance weighing (IDW) techniques

Climate variables essential for agro-hydrological applications are typically recorded at meteorological stations, with the data representing only the specific locations where

measurements are taken. Spatial interpolation techniques are employed to estimate these variables at other locations, enabling broader spatial analysis and application. In this study, station-based measurements of growing season mean air temperatures, and total precipitation, were interpolated using the inverse distance weighting (IDW) method in ArcMap 10.2's Spatial Analyst Toolset. IDW is a deterministic interpolation technique that assigns weights to data points based on their proximity to the prediction location, with nearby points having greater influence than those farther away. The algorithm followed by the IDW interpolation technique to determine the value of the variable of interest at unknown location $M(S_O)$ is:

$$M(S_O) = \sum_{i=1}^{N} \gamma_i \, M(S_i)$$

where, S_O is the location at which the value is to be predicted and $M(S_O)$ is the value for the prediction location S_O, Si is the ith location and $M(S_i)$ is the known value at the ith location, γ_i is an unknown weight for the known value at the ith location.

$$\gamma_i = \frac{d_i^{-p}}{\sum_{i=1}^{N} d_j^{-p}}$$

where, N is the total number of known points, d is the distance of the unknown value location from the known value location, and p is a power parameter controlling the weight distribution. The significance of the power parameter (p) is that its magnitude governs the assignments of weights to the points. Higher values of p concentrate weight on closer points, creating a less smooth surface, while lower p values result in a smoother surface. For this study, the optimal p value was determined using ArcGIS 10.2.

C2. Validations

Comparison and validation of the gridded mean temperature and precipitation data were conducted using independent weather station observations at a monthly timescale for the period 2010–2018 (Supplementary Table 5). Due to the limited number of meteorological gauging stations within and near the lowland crop-growing regions of the country (see Fig. 1 in the main text), validation was performed using a subset of stations randomly selected from a relatively dense network, comprising approximately 10% of the total stations.

A point-to-pixel comparison approach was employed, where gridded climate data were evaluated against corresponding station observations. In this method, the mean temperature and precipitation values from each grid cell were compared to the observed data from the weather station located within the same grid cell [2].

Table 5. Comparison and validation of monthly simulated climate datasets with the observed dataset during 2010−2014.

Climate variables	Maximum differences	Minimum differences	Mean differences	Pearson correlation coefficient (CC)	Mean absolute error (MAE)	Root mean square error (RMSE)
Mean temperature (oC)	5.49	-0.001	0.24	0.95	0.035	0.12
Precipitation (mm)	224.19	-0.008	63.69	0.91	2.13	15.85

The performance evaluation of the simulated climate datasets for the period 2010–2014 showed a strong overall agreement with the observed data. Simulated mean temperature demonstrated high accuracy, evidenced by a strong Pearson correlation coefficient (0.95), low mean absolute error (MAE = 0.035°C), and root mean square error (RMSE = 0.12°C), indicating minimal bias and error. The precipitation dataset also performed well (CC = 0.91), though it exhibited greater discrepancies, as indicated by a higher mean difference (63.69 mm) and RMSE (15.85 mm). These results confirm the robustness of the temperature simulations and underscore the greater challenges involved in accurately reproducing precipitation patterns, particularly extreme events, within gridded climate datasets.

D. Trends and covariation estimation

D1. Coefficients of variation calculation

To assess interannual variability, the coefficients of variation (CVs) for climatic factors and crop yield over the study period (1995–2018) were calculated. The CV provides a normalized measure of variability relative to the mean. For each selected administrative zone and district, the average CV was calculated as the ratio of the standard deviation to the mean for growing season average temperature (T) or total precipitation (P). These CVs were derived using aggregated data extracted from gridded datasets for T and P. Similarly, the average CV for crop yields was calculated as the ratio of the standard deviation to the mean yield at the administrative zone or district level. A higher CV value indicates greater spatial variability, while a lower CV reflects more consistency across the region.

$$CV(X_i) = \frac{Standard\ deviation\ (X_i)}{Mean\ (X_i)}$$

where X_i is T or P for each gauge station at station i.

D2. Linear least square regression method

Least squares linear regression method were also applied to further evaluate trends and correlations between crop yield and climate variables.

$$Y_i = a + bX_i$$

where Y_i is the response, a is the constant coefficient for the trend, b is the slope for the trend and X_i crop yield and climate variable at each administration zone (AZ) and district.

E. First difference regression model

To evaluate the relationship between the time series for yield and climate and compare with the mixed-effects regression model, we used a common approach [3, 4] based on the first-difference time series for yield and climate (i.e. the difference in values from one year to the next). The use of first differences minimizes the influence of slowly changing factors such as crop management and technological improvements. We performed multiple linear regressions with first differences in yield (Y) as the response variable, and first differences of growing season mean temperature (T), precipitation (P) and solar radiation (R) as predictor variables. The following model employed to evaluate the first-difference impact of yield on year-to-year changes in T, P, and R for each administrative zone included the intercept of the regression, representing the average annual yield change with climate conditions held constant.

$$\Delta Log\ Y_i = \beta_o + \beta_t \Delta T_i + \beta_p \Delta P_i + \beta_r \Delta R_i$$

Where $\Delta Log\ Y_i$, the first difference in log is yield of administrative zone i, β_o is the intercept and β_t, β_p, β_r represent the coefficient of crop yields to temperature, precipitation, and solar

radiation, respectively. ΔT_i is the change in mean temperature (°C), ΔP_i is the change in precipitation (mm), and ΔR_i is the change in solar radiation (W/m^2).

The resulting regression coefficients imply the percent of change in yield per unit of weather change or sensitivity (e.g., the % change in yield per 1 °C of T or 1mm of P increase). These coefficients were then multiplied by the weather trends (e.g., °C per year) observed during the study period (1995 to 2018). This computation allowed us to determine the impact of individual and combined weather variables on yield trends at the administrative zone level.

F. Mixed-effects model implementation

To assess the effects of climatic trends on crop yields, we utilized a mixed-effects regression model to quantify the relative influence of climate variables on yield variability for four crops: maize, sorghum, tef, and wheat. These analyses were conducted at the administrative zonal and district levels. Given the spatial dependence of crop and climate data, we incorporated administrative zones or districts as random effect variables. Fixed effect variables included year, temperature (T), precipitation (P), and their quadratic terms (T^2 and P^2).

The model accounted for the hierarchical structure of the data, recognizing that repeated observations within the same administrative zone over time are more closely related than observations from different zones. Employing simple linear regression in such cases would breach the statistical assumption of independent observations. To address this issue, we incorporated a first-order autoregressive (AR1) structure to account for temporal autocorrelation within the dataset. Additionally, a random effects covariance structure was included to model correlations between random intercepts and slopes for temporal trends and climatic variables.

The Akaike Information Criterion (AIC) [5] was employed to identify the best-fitting model for each crop. The performance of the selected model was further validated using a t-test at a significance threshold of p = 0.05, ensuring its statistical superiority over alternative mixed-effects models.

$$Log(Y_{i,t}) = \beta_0 + b_{0i} + (\zeta_i + z_i) * year + (\beta_{j,i} + b_{j,i}) \cdot X_{j,i,t} + \varepsilon_{i,t}$$

where $Log(Y_{i,t})$ represents the logarithm of yield for AZ i in year t, β_0 is the global intercept, b_{0i} is the random intercept for each AZ, z_i is the random slope for the temporal trend

(year) that adds variation to the fixed effect, ζ_i, of time in the AZs, the vector $\beta_{j,i} + b_{j,i}$ contains coefficients for the jth climatic predictor variables $X_{j,i,t}$ (i.e. T, T^2, P, P^2, and R), composed of a fixed effect representing the average response to the climatic variable, $\beta_{j,i}$, and a random effect, $b_{j,i}$, accounting for the variability in the effect of climate in the AZs, and $\varepsilon_{i,t}$ is the error term, which was assumed to have an AR1 structure that allows identifying temporal autocorrelation within the AZs.

$$\varepsilon_{i,t} = \rho\varepsilon_{i,t-1} + \eta_{i,t}$$

where ρ is autoregressive parameter, and η is independent noise term.

All variables are standardized to z-scores by subtracting their mean and dividing by their time-series standard deviation. This transformation enables direct comparison of the magnitudes of each β coefficient.

In order to mitigate potential non-normal yield distributions, we employed a logarithm transformation (log yields) (see Supplementary Fig. 10 below). Incorporating linear time terms allowed us to remove the trend from yield data, thereby accommodating the influence of factors beyond weather (such as technological advancements). Moreover, the inclusion of quadratic terms for temperature and precipitation accounts for the typical phenomenon where in crops demonstrate an optimal range for these variables, beyond which yields tend to decrease [6, 7]. In this mixed-effects model, the intercepts and slopes vary randomly across locations, capturing unobserved heterogeneity. The random intercepts and slopes associated with growing areas (administrative zones or districts) and time are intended to account for the variance induced by location-specific omitted variables within the model, such as soil quality, crop management practices, and other technological advancements [6].

Temperature and precipitation are key climatic factors for crop growth and development, influencing various physiological and phenological stages. While their effects on yield may exhibit non-linear patterns, the linear components capture essential baseline trends in crop responses to these variables. According to the principle of marginality, higher-order terms such as T^2 and P^2 cannot be included in a model without their corresponding lower-order terms (T and P). Excluding these linear terms would result in biased coefficient estimates for the quadratic terms, potentially leading to misinterpretation of their effects. While T and P may not always show strong statistical significance in this mixed-effects model, their inclusion ensures

the model remains mathematically sound, biologically relevant, and interpretable, while maintaining the hierarchical structure necessary for accurate inference.

We then used the multiple linear regression model (F (T, P, R)) to compute:
(I) F(T, P, R) = **predicted yields** with observed weather,
(II) F(Td, P, R) = **predicted yields** with detrended temperature, observed precipitation and observed radiation,
(III) F(T, Pd, R) = **predicted yields** with observed temperature, detrended precipitation and observed radiation,
(IV) F(T, P, Rd) = **predicted yields** with observed temperature, observed precipitation and detrended radiation, and
(V) F(Td, Pd, Rd) = **predicted yields** with detrended weather

Finally, we computed the trends of the differences to quantify the yield effect of trends in temperature, precipitation, and solar radiation both in individually and combination.

- Relative **temperature trend effect** to the overall yield variability = ((Y(I) – Y (II))/ Y(I))*100
- Relative **precipitation trend effect** to the overall yield variability = ((Y(I) – Y (III))/ Y(I))*100
- Relative **solar radiation trend effect** to the overall yield variability = ((Y(I) – Y (IV))/ Y(I))*100
- Relative **climate trends effect** to the overall yield trends = ((Y(I) – Y (V))/ Y(I)) *100

A sensitivity analysis was performed by incorporating minimum and maximum temperatures as separate covariates in the model (see Supplementary Fig. 11 below). The results from this alternative specification aligned closely with those based on mean temperature, indicating consistency across model formulations.

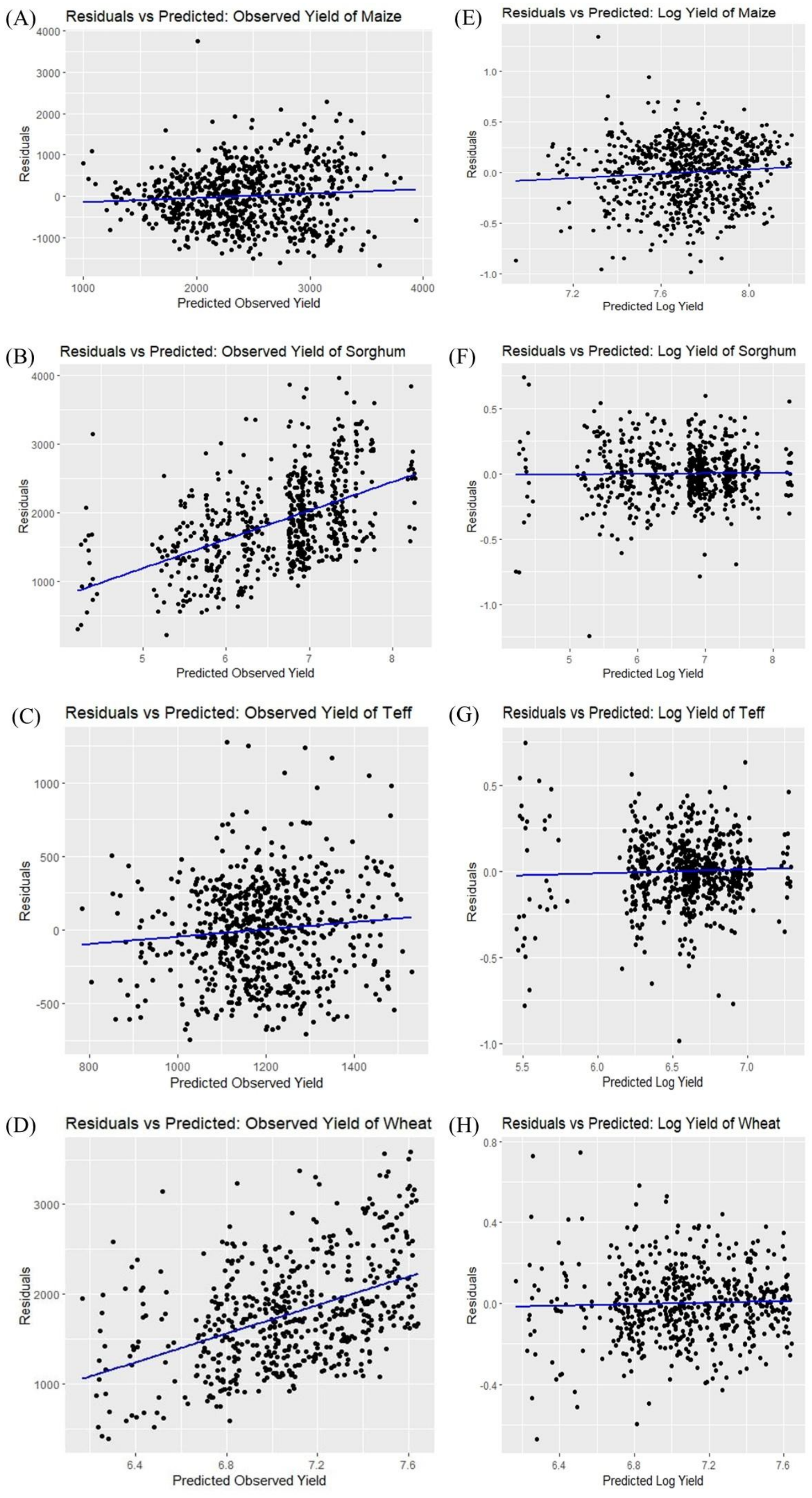

Fig. 10. Scatter plots showing residuals versus predicted yield for observed (A–D) and transformed (E–H) data across different crops: maize (A, E), sorghum (B, F), tef (C, G), and wheat (D, H).

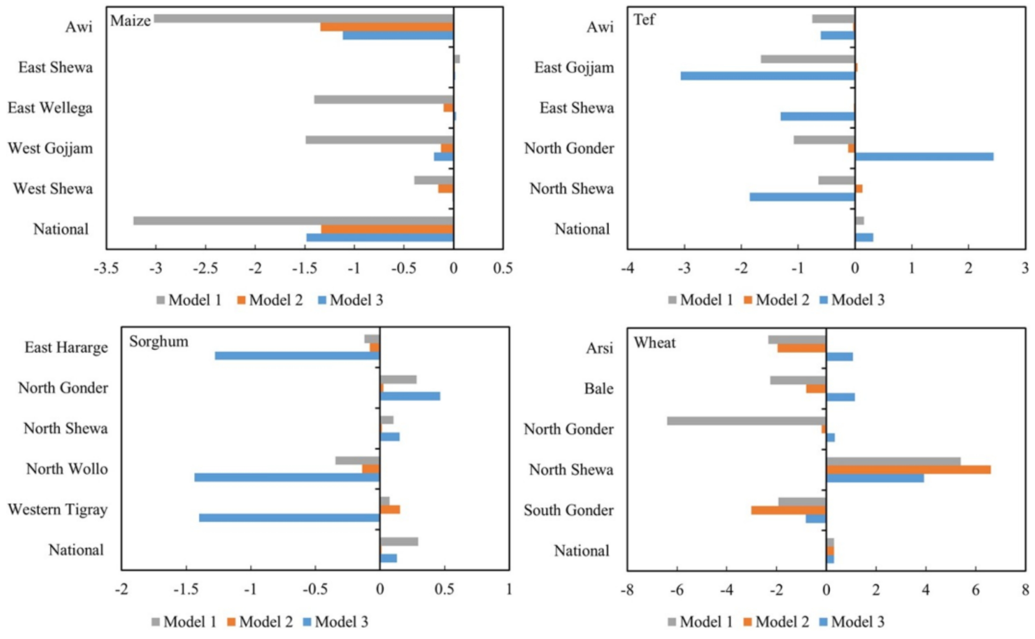

Fig. 11. Comparison of different model specifications using separately modeled temperature variables: mean temperature (TMEAN), maximum temperature (TMAX), and minimum temperature (TMIN). Model 1: Mixed-effects model with TMEAN modeled separately (main model used in the paper). Model 2: Mixed-effects model with TMAX modeled separately instead of TMEAN. Model 3: Mixed-effects model with TMIN modeled separately instead of TMEAN.

References

1. Central Statistical Agency (CSA). Agricultural Sample Survey 2018/2019 (2011 E.C.), Vol. 1: Report on Area and Production of Major Crops (Private Peasant Holdings, Meher Season). *Statistical Bulletin* 589, Addis Ababa, Ethiopia (2019).
2. Gebere, S. B., Alamirew, T., Merkel, B. J. & Melesse, A. M. Performance of high resolution satellite rainfall products over data scarce parts of eastern Ethiopia. *Remote Sens.* **7**, 11639–11663 (2015).
3. Lobell, D. B. *et al.* Analysis of wheat yield and climatic trends in Mexico. *Field Crops Res.* **94**, 250–256 (2005).
4. Nicholls, N. Increased Australian wheat yield due to recent climate trends. *Nature* **387**, 484–485 (1997).
5. Akaike, H. A new look at the statistical model identification. *IEEE Trans. Autom. Control* **19**, 716–723 (1974).
6. Lobell, D. B., Schlenker, W. & Costa-Roberts, J. Climate trends and global crop production since 1980. *Science* **333**, 616–620 (2011).
7. Jones, J. W. *et al.* The DSSAT cropping system model. *Eur. J. Agron.* **18**, 235–265 (2003).
8. Warner, J., Stehulak, T. & Kasa, L. Woreda-Level Crop Production Rankings in Ethiopia: A Pooled Data Approach. International Food Policy Research Institute (IFPRI), Addis Ababa, Ethiopia (2015).
